# Effectiveness and safety of manual therapy when compared with oral pain medications in patients with neck pain: a systematic review and meta-analysis

**DOI:** 10.1186/s13102-024-00874-w

**Published:** 2024-04-16

**Authors:** Joshua Makin, Lauren Watson, Dimitra V Pouliopoulou, Taylor Laframboise, Bradley Gangloff, Ravinder Sidhu, Jackie Sadi, Pulak Parikh, Anita Gross, Pierre Langevin, Heather Gillis, Pavlos Bobos

**Affiliations:** 1 Comprehensive Musculoskeletal Field, Advanced Health Care Program, School of Physical Therapy, Western University, London, ON Canada; 2 School of Physical Therapy, Health and Rehabilitation Sciences, Western University, London, ON Canada; 3 Western’s Bone and Joint Institute, Collaborative Musculoskeletal Health Research Program, London, ON Canada; 4 School of Rehabilitation Sciences, McMaster University, Hamilton, ON Canada; 5 School of Rehabilitation Sciences, Université Laval, Quebec City, QC Canada; 6 School of Physical and Occupational Therapy, McGill University, Montréal, Canada; 7 Physio Interactive, Quebec City, QC Canada

**Keywords:** Manual therapy, Neck pain, Effectiveness, Safety, Systematic review, Meta-analysis

## Abstract

**Background:**

This systematic review and meta-analysis seeks to investigate the effectiveness and safety of manual therapy (MT) interventions compared to oral pain medication in the management of neck pain.

**Methods:**

We searched from inception to March 2023, in Cochrane Central Register of Controller Trials (CENTRAL), MEDLINE, EMBASE, Allied and Complementary Medicine (AMED) and Cumulative Index to Nursing and Allied Health Literature (CINAHL; EBSCO) for randomized controlled trials that examined the effect of manual therapy interventions for neck pain when compared to medication in adults with self-reported neck pain, irrespective of radicular findings, specific cause, and associated cervicogenic headaches. We used the Cochrane Risk of Bias 2 tool to assess the potential risk of bias in the included studies, and the Grading of Recommendations, Assessment, Development, and Evaluations (GRADE) approach to grade the quality of the evidence.

**Results:**

Nine trials (779 participants) were included in the meta-analysis. We found low certainty of evidence that MT interventions may be more effective than oral pain medication in pain reduction in the short-term (Standardized Mean Difference: -0.39; 95% CI -0.66 to -0.11; 8 trials, 676 participants), and moderate certainty of evidence that MT interventions may be more effective than oral pain medication in pain reduction in the long-term (Standardized Mean Difference: − 0.36; 95% CI − 0.55 to − 0.17; 6 trials, 567 participants). We found low certainty evidence that the risk of adverse events may be lower for patients that received MT compared to the ones that received oral pain medication (Risk Ratio: 0.59; 95% CI 0.43 to 0.79; 5 trials, 426 participants).

**Conclusions:**

MT may be more effective for people with neck pain in both short and long-term with a better safety profile regarding adverse events when compared to patients receiving oral pain medications. However, we advise caution when interpreting our safety results due to the different level of reporting strategies in place for MT and medication-induced adverse events. Future MT trials should create and adhere to strict reporting strategies with regards to adverse events to help gain a better understanding on the nature of potential MT-induced adverse events and to ensure patient safety.

**Trial registration:**

PROSPERO registration number: CRD42023421147.

**Supplementary Information:**

The online version contains supplementary material available at 10.1186/s13102-024-00874-w.

## Background

Neck pain is a highly prevalent musculoskeletal disorder that is a major cause of morbidity and disability worldwide. Globally in 2017 the age standardized rates for point prevalence of neck pain was 3551.1 per 100,000 population, the age standardized rates for incidence of neck pain was 806.6 per 100,000 population, and the age standardized rates for years lived with disability from neck pain was 352.0 per 100,00 population [1].

Most episodes of acute neck pain will resolve within 2 months, but nearly 50% of individuals will continue to experience some degree of pain or frequent re-occurrences [2]. Amongst the most commonly used conservative treatments are oral pain medications, and manual therapy (MT) [3, 4]. Oral pain medications include oral analgesics, nonsteroidal anti-inflammatory drugs (NSAIDs) and opioids, and they are frequently used to mitigate neck pain [3, 5, 6]. Current clinical practice guidelines suggests a weak positive recommendation for oral analgesics (including medication for neuropathic pain) and specifically paracetamol, NSAIDS (short-term only), and opioids including tramadol (short term only) [7]. However, recent systematic reviews have reported limited or inconclusive evidence on NSAIDs short and long-term effect [8, 9]. In addition, a recent randomised trial of 347 patients that compared the efficacy of guideline-recommended care plus an opioid (oxycodone-naloxone, up to 20 mg oxycodone per day orally) to guideline-recommended care and an identical placebo in patients with non-specific low back or neck pain found no difference in the mean pain score on the short-term follow-up (6 weeks) between the two groups [10]. Research also suggests that oral medications are not well-tolerated by everyone and carry a risk of various adverse events such as gastrointestinal issues, respiratory depression, fractures, myocardial infraction and opioid dependence [11, 12].

MT is a non-pharmacological intervention utilizing hands-on techniques like joint mobilization, manipulation, and soft tissue massage. These techniques aim to enhance joint and muscle function, reduce pain, and improve overall well-being [6, 13, 14]. Proposed mechanisms for MT’s beneficial effects include enhancing joint and muscle function, boosting local blood flow, and reducing inflammation [15]. Furthermore, MT may exert neurophysiological effects, thereby reducing pain and enhancing physical function, offering an alternative for treating musculoskeletal pain without medications [15, 16].

Despite the widespread use of oral medication, the evidence on their effectiveness in managing neck pain remains controversial. Combined with potential adverse associated with oral medications such as NSAIDs and opioids, MT may present a safer, more favorable treatment option for neck pain. Thus, this systematic review and meta-analysis seeks to investigate the effectiveness and safety of MT compared to oral pain medication in the management of neck pain.

## Methods

We adhered to the Preferred Reporting Items for Systematic Reviews and Meta-Analysis (PRISMA) guidelines in search strategy and reporting [17]. The study was registered in PROSPERO database with registration number: CRD42023421147.

### Search strategy

We searched from inception to March, 2023, in Cochrane Central Register of Controller Trials (CENTRAL), MEDLINE, EMBASE, Allied and Complementary Medicine (AMED) and Cumulative Index to Nursing and Allied Health Literature (CINAHL; EBSCO). Example of search terms that were utilized were “Mulligan”, “Maitland”, “Kaltenborn”, “manipulation”, “mobilization”, “Manual therapy”, and “neck pain”. The full search strategy is included in Additional File 1. To identify additional eligible studies, we reviewed the reference list of all included trials, searched clinical trial registries (ClinicalTrials.gov and WHO International Clinical Trials Registry Platform) for trials in progress, and examined the reference lists of previously published systematic reviews. No language restrictions were imposed.

### Eligibility criteria

We included only randomized trials with participants aged 18 and older experiencing neck pain with or without radicular findings, either non-specific, due to a whiplash-associated disorder (WAD categories I and II) or associated with cervicogenic headaches. Acute symptoms were defined as lasting less than 30 days, subacute as 30–90 days, and chronic as longer than 90 days. Trials were grouped by the symptom duration of the majority (> 80%) of participants. The included interventions were manual therapies (MT) such as mobilization and/or manipulation of the cervical spine. Comparator groups consisted of those receiving oral or topical medications (NSAIDs, acetaminophen, opioids, topical anti-inflammatory creams). Trials with usual care arms were also included if they prescribed medication as part of the usual care and they did not include a manual therapy component. We excluded non-randomized studies, or studies with participants displaying potential long tract signs, neck pain due to pathologies such as cancer, or studies with any use of intra-articular treatments or other injections. The primary outcomes of interest were neck pain at short term (directly post intervention) and long term (6–12 months follow-up); and adverse events (AEs) and serious adverse events (SAEs) resulting from manual therapy and medication treatments. AE’s are defined as the ramification of MT with moderate symptoms such as increased pain, tiredness, stiffness, spasm, headaches and dizziness which resolve over time [18]. SAEs are defined as death and stroke, occurring secondarily to a cervical vascular dissection [19, 20]. The secondary outcome was all-cause dropouts. We used dropout ratios to further explore the safety of the included interventions by assessing tolerability and to account for potential treatment discontinuation rates attributed to non-reported adverse events.

### Study selection

Two pairs of reviewers (LW/JM, TL/BG) independently assessed each article in a two-stage process, title/abstracts and full texts. Potential articles were identified using a pre-defined search strategy and imported into Covidence for initial title and abstract screening (AG/JP). Any disagreements in the title and abstract screening underwent full review. Any disagreements in the full text stage were resolved by a separate third reviewer from the initial group.

### Data extraction

The same set of reviewers extracted the following information: authors’ names, publication year, sample size, demographic details, gender distribution, neck pain type, intervention, comparator, outcomes of interest (including pain intensity and adverse events), follow-up duration (extracted in weeks), funding source, potential conflicts of interest. The extracted data were entered in duplicate into Covidence and cross-verified by a third reviewer (DVP).

The primary outcomes of interest were neck pain and AEs (including SAEs). The secondary outcome was all-cause drop-out rates. For pain outcomes, we extracted data at baseline, short-term follow-up (directly post in-intervention) and long term follow up (6-12months post intervention). If a trial reported more than one pain outcome measure, we prioritized the Visual Analogue Scale (VAS), or the Numeric Pain Rating Scale. If a trial reported both of these scales, or none of these scales, we prioritized the scale that was reported as the primary outcome of interest. Data on adverse events (AEs) and serious adverse events (SAEs) and data on all-cause dropouts were gathered at the latest available timepoint.

We also used the TiDier checklist to assess the quality of reporting of each trial’s individual components.

### Risk of bias

To assess the potential risk of bias in the included studies, we used the Cochrane Risk of Bias 2 tool [21]. This tool considers multiple domains including the randomization process, deviations from the intended interventions, missing outcome data, and the selection of the reported results. Two independent reviewers (RS, LW) carried out the risk of bias assessment for each trial, with any discrepancies being resolved by a third reviewer (PB). We used the Cohen’s kappa to calculate the level of agreement among reviewers.

### Data analysis

We employed a random-effects Sidik-Jonkman model for the analysis of pain outcomes across trials, and a fixed-effects Mantel-Haenszel model was used for the analysis of all cause dropouts, adverse and serious adverse events. Given our assumption of potential variability amongst the treatment effects of different trials, a random-effects model was chosen. We expressed the pain outcomes as standardized mean differences (SMDs) of change scores, complemented by 95% confidence intervals (CI). To further aid in the clinical interpretation of our results, we used the benchmarks suggested by Cohen [22] to interpret our effect sizes to small (SMD = 0.2), moderate (SMD = 0.5), and large (SMD = 0.8) and we have indicated dashed lines in our forest plots representing a between-group minimal important difference (MID) threshold of 0.4 SD [23].We calculated risk ratios (RRs) with 95% CI for the analysis of adverse events, treating them as dichotomous outcomes. Risk Ratios were considered large if RR > 2 or < 0.5 and very large if RR > 5 or < 0.2. We used dash lines in our forest plots to mark the 20% between group difference for adverse events to indicate a clinically meaningful threshold [24]. We examined statistical heterogeneity using the τ^2^ (between trial variance) [25] and I^2^ statistic. An I² estimate of at least 50%, was used to interpret for evidence of a substantial heterogeneity [26]. We applied the Grading of Recommendations, Assessment, Development, and Evaluations (GRADE) approach to grade the quality of the evidence and strength of recommendations identified in the included trials and we assigned a certainty rating (high, moderate, low, or very low) to each outcome across trials, representing our confidence level in the effect estimates [27]. Based on the GRADE guidelines, we evaluated the quality of evidence based on four criteria:1) study limitations or risk of bias), 2) publication bias, 3) imprecision, 4) indirectness, and 5) inconsistency [27].

Risk of bias: If an individual trial (per outcome) was rated as “low risk” of bias or “unclear risk” of risk of bias (with low risk of bias for randomization, allocation concealment, and blinding domains), we considered there was no serious study limitations and did not downgrade the quality of evidence. If an individual trial (per outcome) was rated as “high risk” of bias (with high risk of bias only for incomplete outcome data, selective reporting or other bias), we considered this as a serious limitation and downgraded the quality of evidence by one level. Lastly, if an individual trial (per outcome) was rated as “high risk” of bias (with high risk of bias for randomization, allocation concealment or blinding domains), we considered this as a very serious limitations and downgraded the quality of evidence by two levels [28].

Publication bias: In the presence of publication bias, we planned to assess by utilizing funnel plots and impute the missing studies to account for publication bias in the meta-analysis [29].

Imprecision: The judgement of the quality of evidence regarding imprecision was based on the Optimal Information Size (OIS) calculation [30]. An OIS of approximately 400 (200 per group) was calculated based on the usual standards of α (0.05) and β (0.20) and an effect size of 0.2 standard deviations, indicating a small effect. If the OIS criterion was not met, we downgraded the level of evidence by one level for imprecision (i.e., serious imprecision).

Indirectness: The GRADE assessment of indirectness was based on differences in outcome measures (patient-important outcomes vs. surrogate outcomes). In this review, we only included patient-important outcomes. Therefore, we did not downgrade the quality of evidence if RCTs reported patient-important outcomes (pain and adverse events) [31].

Inconsistency (statistical): To assess inconsistency we considered a τ^2^ = 0.10 or greater or I^2^ = 40% or greater as evidence of a moderate heterogeneity, and therefore, we downgraded by one level [32].

To enhance the clarity and completeness of reporting on the interventions studied given the potentially multimodal nature of manual therapy we used the TiDier Checklist [33], and we conducted a sensitivity analysis based on the individual components of the interventions of the included trials.

### Publication bias

The potential of publication bias relating to small study sample size was assessed by applying the Regression based Egger test [34], and we imputed the missing studies to compensate for this bias within the meta-analysis. The non-parametric “trim and fill” method allowed us to compare the observed and imputed studies. To determine if imputed studies fell within the region of statistical significance, we used contour-enhanced funnel plots [35]. All analyses were performed using STATA (Stata Statistical Software: Release 17, StataCorp LLC).

## Results

**Fig. 1:**
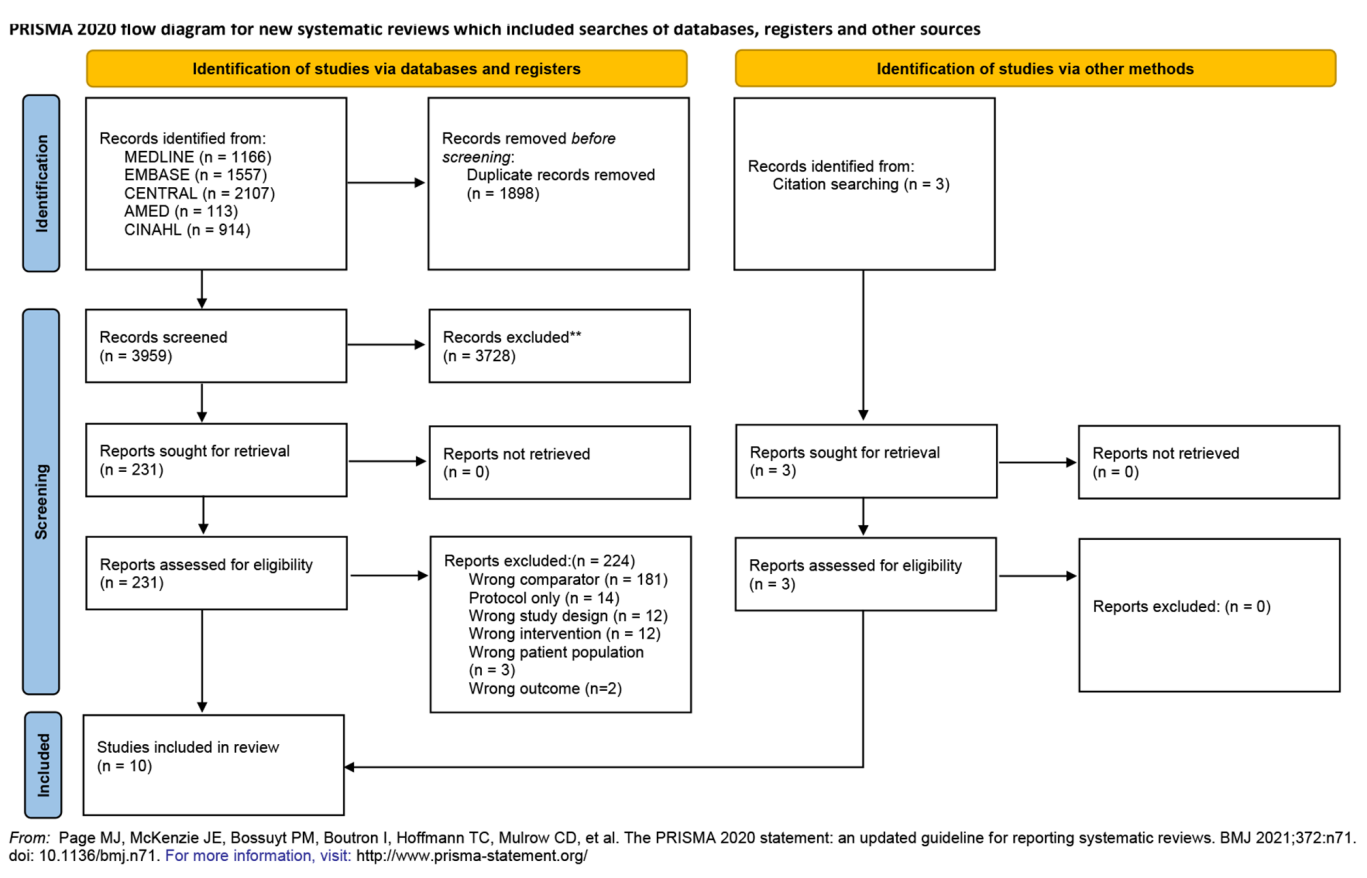
PRISMA flow diagram

The PRISMA flow diagram detailing the selection process is shown in Fig. 1. We identified a total of 5,857 records. After duplicates were removed, we screened 4,197 studies in title/abstract level. A total of 233 were assessed at full text level and a total of 7 records were eligible. From reviewing the eligible papers references, we were able to add 3 more records. This brought the total to 9 trials (10 records) which met all the pre-determined inclusion criteria. A list of the studies that were excluded at full-text level with the reasons for exclusion are provided in Additional File 2.

### Study characteristics

Table 1 presents the individual study descriptive characteristics, intervention and comparator details and primary outcomes. Nine trials with a total of 779 participants were included in the meta-analysis. The sample size in the included studies ranged from 37 to 191. The median total sample size was 84.5 participants (IQR 51 to 124). All trials were conducted on adults over the age of 18 years. The median average age of participants was 45 (IQR 40.5 to 46.5). The median percentage of females was 59.5% (IQR 56-68%).

**Table 1:** Summary of subject and study descriptive characteristics, intervention and comparator arms

Author	Country	Age	N (% males)	Primary Outcomes	Follow up (weeks)	Intervention Details	Comparator Details	
Brontford et al. 2012 [36]	United States	47.55 (mean)	191 (63.1)	NPRS Pain Score	2, 3, 8, 12, 26, 52 weeks	Manipulation/mobilization of hypomobile segments	NSAIDS, acetaminophen, opioid, muscle relaxant	
Calvo-Lobo et al. 2018 [41]	Venezuela	32.05 (mean)	51 (21)	NPRS Pain Score	1, 15, 30 weeks	Non-invasive neural mobilization technique using a cervical contralateral glide mobilization	Ibuprofen	
De Hertogh et al. 2009 [37]	Belgium	43.22 (mean)	37 (9)	VAS pain score	7, 12, 26 weeks	Manual therapy (articular mobilisations and low load exercises)	Usual Care/Medication (NSAIDS, triptans)	
Giles et al. 1999 [38]	Australia	38.75 (median)	57 (23)	VAS pain score	4 weeks	Manual Therapy SMT (high velocity/low amplitude spinal manipulation)	Medication (NSAID)	
Giles et al. 2003 [40]	Australia	39 (median)	75 (31)	VAS pain scale	Initial visit, 2, 5 and 9 weeks	Spinal manipulation	Celebrex (200-400 mg/day), Vioxx (12.5-25 mg/day), paracetamol (up to 4 g/day)	
Hoving et al. 2002 [44]	Netherlands	45.25 (mean)	124 (53.9)	NPRS pain score	3, 7	Manual Therapy (Muscular and articular mobilizations, coordination and stabilization techniques)	Continued Care (paracetamol or nonsteroidal anti- inflammatory drugs)	
Hoving et al. 2006 [39]	Netherlands	45.25 (mean)	124 (53.9)	NPRS pain score	3, 7, 13, 26, 52	Manual Therapy (Muscular and articular mobilizations, coordination and stabilization techniques)	Continued Care (paracetamol or nonsteroidal anti- inflammatory drugs)	
Lee et al. 2021 [42]	Korea	38.4 (mean)	108 (35)	VAS & NPRS pain score	5, 12.9, 25.8, 38.7, 51.6 weeks	Chuna manual therapy (Manipulation/mobilization using distraction & adjustment methods)	Usual care (PT + oral medications - Analgesics (paracetamol or NSAID))	
Muller et al. 2005 [57]	Australia	39 (median)	42 (21.99)	VAS pain score	9 & >103.2 weeks	Manipulation (left to discretion of provider)	Medication (Celecoxib, rofecoxib, acetaminophen)	
Walker et al. 2008 [43]	United States	47.5 (mean)	94 (31)	VAS pain score	3, 6, 103.2 weeks	Manual therapy and Exercise (1/3 Thrust or Non-thrust, muscle energy, stretching)	Usual Care (general practitioner care + instructions to continue prescription medication– NSAIDs, Cervical ROM exercises)	

There was a mix of different MT interventions in the included studies. Five trials used manipulation and mobilization of hypomobile segments and muscular/articular mobilizations [36–40]. One trial used non-invasive neural mobilization techniques [41]. One trial used manipulation and mobilization techniques using distraction and adjustment methods (Chuna manual therapy) [42]. One trial used a combination of articular mobilization with low load exercise [37]. One study used a combination of MT plus a standardized exercise program [43]. One study used a combination of MT with coordination and stabilization techniques [39, 44]. Details about the interventions are found in Additional File 3. The comparator groups were oral medication, usual care or continued care. The medications were NSAIDS, acetaminophen, opioids, muscle relaxants, paracetamol [36–39, 41, 42, 44]. One usual care group included physiotherapy plus oral medication [42]. One included medication with home exercise and advice on staying active [36].

Eight trials (676 participants) contributed to the short-term analysis for pain. The short-term follow-up ranged from 3 to 12 weeks (median 6.5; IQR 4.75 to 9.74). Six trials (567 participants) contributed to the long-term analysis for pain. The long-term follow up ranged from 36 weeks (1 trial) to 52 weeks (5 trials). Two pain rating scales were utilized in the included studies, the Visual Analogue Scale (VAS) and the Numeric Pain Rating Scale. Eight trials (745 participants) reported data for all-cause dropouts, and 5 trials (426 participants) reported estimates for adverse events. A detailed analysis on the quality of the reporting of each trial’s individual components is summarized using the TiDier checklist in Additional File 4.

### Risk of bias

We found low risk of bias in the randomization process in 6 trials (79%, *n* = 664) that reported pain and 2 trials (34%, *n* = 284) that reported safety outcomes; low risk of bias owing to deviations from intended interventions in 8 trials (84%, *n* = 704) that reported pain and 4 trials (38%, *n* = 324) that reported safety outcomes; low risk of bias due to missing outcome data in 3 trials that reported pain (40%, *n* = 338); and low risk of bias in the selection of reported results in 8 trials that reported pain (87%, *n* = 735) and in 3 that reported safety (38%, *n* = 319). None of the included trials was rated as having low risk of bias for the measurement of the outcome, hence no trial was found to have low risk of bias overall. Details about the risk of bias are summarized in Additional File 5. The Cohen’s kappa was 0.9 suggesting a very high level of agreement between the reviewers.

### Publication bias

Egger’s regression test and visual inspection of the contour enhanced funnel plot [23, 24] symmetry revealed no small-study effect for short-term pain, long term pain and adverse events. Funnel plots are included in Additional File 6, 7.

### Pain level reduction in MT and medication

**Fig. 2:**
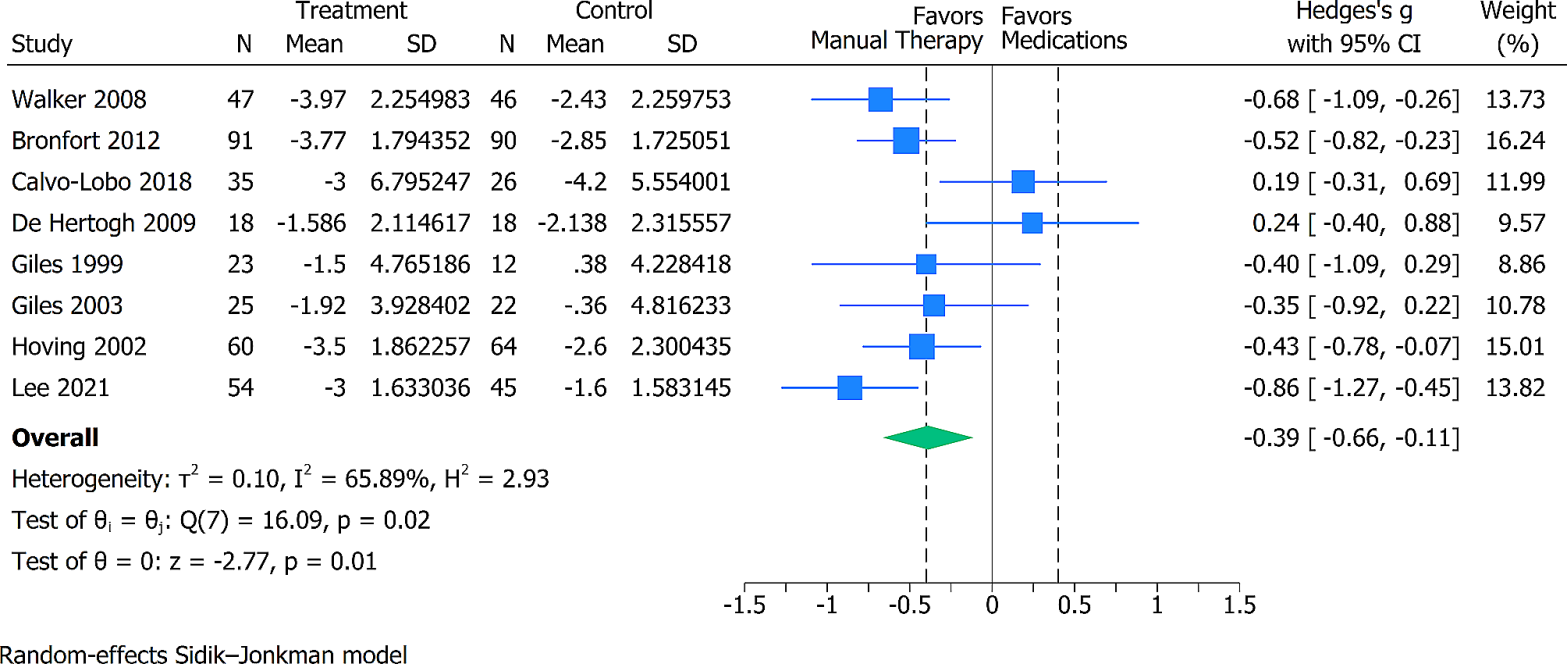
Forest plot showing the SMD with 95% CI for between group comparisons of short-term pain (up to 12 weeks). The dotted lines represent a between-group minimal important difference (MID) threshold of 0.4 SD

For the short-term pain intensity 8 trials (676 participants) were analyzed. Compared with the oral pain medications, the pooled effects estimate demonstrated moderate effects on pain intensity reduction in favor of the intervention group and this effect was statistically significant (SMD − 0.39; 95% CI -0.66 to -0.11) (Fig. 2). Heterogeneity was high (tau^2^ = 0.10). The certainty of evidence was deemed low, downgraded for high risk of bias and inconsistency (Additional File 8).

**Fig. 3:**
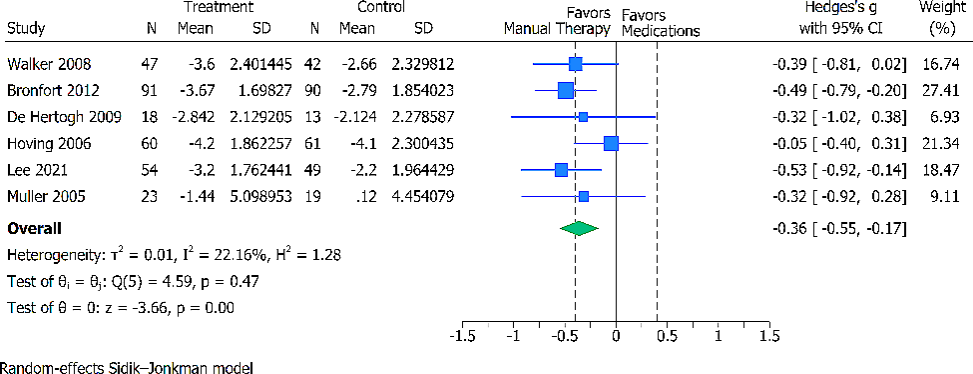
Forest plot presenting the SMDwith 95% CI for between-group comparisons of long-term pain (25.8 to 51.6 weeks). The dotted lines represent a between-group minimal important difference (MID) threshold of 0.4 SD

For the long-term pain intensity 6 trials (567 randomized participants), were analyzed. Compared with the oral pain medications, the pooled effects estimate demonstrated moderate effects on pain intensity reduction in favor of the intervention group and this effect was statistically significant (SMD − 0.36; 95% CI − 0.55 to − 0.17) (Fig. 3). Heterogeneity was low (tau^2^ = 0.01). The certainty of evidence was deemed moderate, downgraded for high risk of bias (Additional File 8).

### Adverse events

**Fig. 4:**
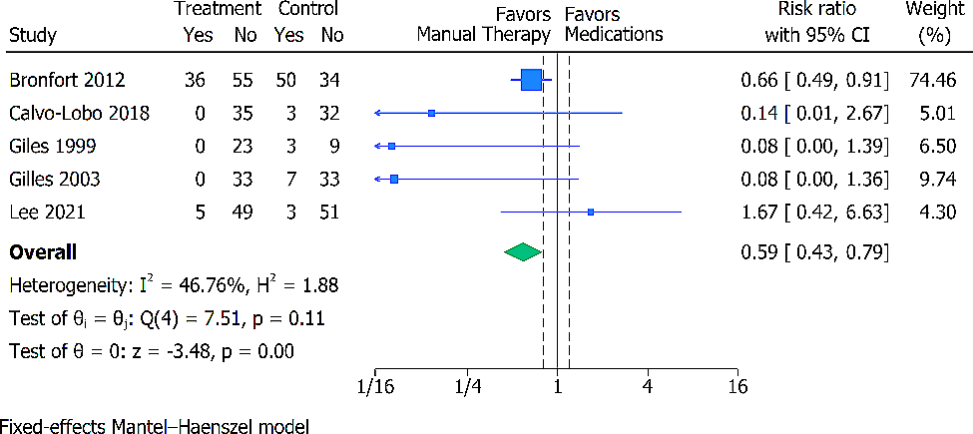
Forest plot presenting the Risk Ratio with 95% CI for between-group comparisons of adverse events. The dotted lines represent a between-group minimal important difference (MID) threshold of 20%

Adverse events were reported in 5 trials (426 participants). There were no timelines for adverse events. The most common adverse event in the MT group was aggravation of pain. The most common adverse events in the oral pain medication groups were GI symptoms, drowsiness, dry mouth, and cognitive symptoms. There were no serious adverse events reported in any of the included studies. When compared with oral pain medications, the pooled effect estimate demonstrated fewer adverse events in favor of the manual therapy group (RR 0.59; 95% CI 0.43 to 0.79) (Fig. 4). Heterogeneity was moderate (I^2^ = 46.76%) The certainty of evidence was deemed low, downgraded for high risk of bias and inconsistency (Additional File 8).

### All cause of dropouts

**Fig. 5:**
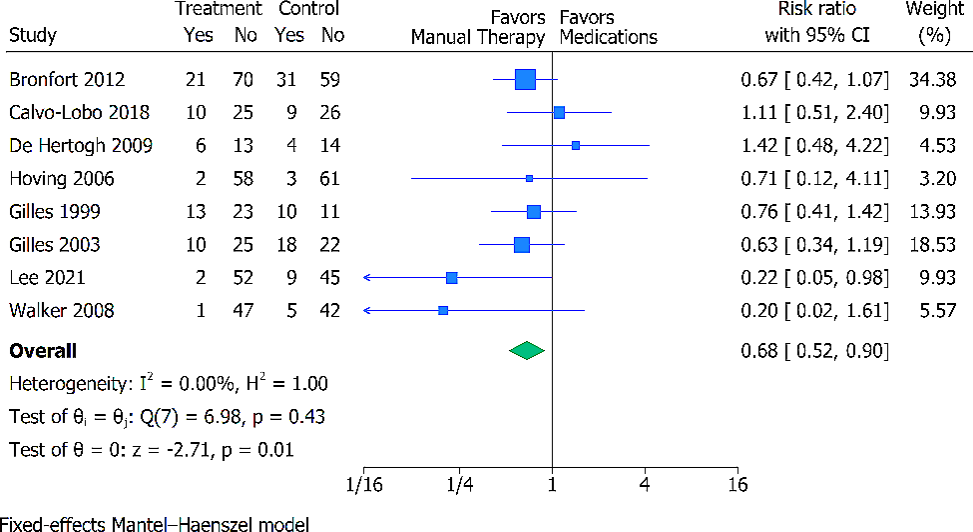
Forest plot presenting the Risk Ratio with 95% CI for between-group comparisons of all cause drop-outs

For the all-cause dropouts, 8 trials (745 participants) were analyzed. The pooled effect estimate indicated that participants who received pain medications were associated with a higher risk of all cause of dropouts compared to participants who received MT (RR 0.68; 95% CI 0.52 to 0.90) (Fig. 5). We found no heterogeneity (I^2^ = 0.00%). The certainty of evidence was deemed moderate, downgraded for high risk of bias (Additional File 8).

### Sensitivity analysis

Six of the included studies used MT as a standalone treatment and 3 used MT with an exercise component. Hence, to provide a more comprehensive analysis we conducted a subgroup analysis on the effectiveness of MT in short-term and long-term pain reduction based on the presence or absence of an exercise component as an adjunct to MT.

For MT as standalone, the pooled effects estimate demonstrated moderate effects on short-term pain intensity reduction in favor of the intervention group (SMD − 0.41; 95% CI -0.76 to -0.06) compared with the oral pain medications (Additional File 9). For MT plus exercise, the pooled effects estimate demonstrated moderate effects on short-term pain intensity reduction in favor of the intervention group (SMD − 0.34; 95% CI -0.85 to 0.17) compared with the oral pain medications (Additional File 9).

For MT as standalone, the pooled effects estimate demonstrated moderate effects on long-term pain intensity reduction in favor of the intervention group (SMD − 0.48; 95% CI -0.71 to -0.26) compared with the oral pain medications (Additional File 10). For MT plus exercise, the pooled effects estimate demonstrated moderate effects on long-term pain intensity reduction in favor of the intervention (SMD − 0.22; 95% CI -0.5 to 0.07) compared with the oral pain medications (Additional File 10).

## Discussion

In this systematic review with meta-analysis of 9 trials (797 patients) we found low certainty of evidence that MT interventions may be more effective than medication in pain reduction in the short-term, and moderate certainty of evidence that MT interventions may be more effective than medication in pain reduction in the long-term. The effect size for pain reduction did not surpass the pre-defined between-group MID for either short, or long-term pain reduction. All-cause dropouts and risk of adverse events were lower for patients that received MT compared to the ones that received medication. The certainty of evidence was moderate for all-cause dropouts, and low for adverse events. There were no serious adverse events reported in the trials. The effect estimate for adverse events surpassed the pre-defined 20% MID threshold which indicates that MT may have a better safety profile than oral pain medications.

### Previous studies

The results of our study suggest that MT may be more effective than oral pain medications for people with neck pain in both short-term and in the long-term. Our findings are in agreement, and further reinforce the results of recently emerging studies in the neck pain population. With regards to pain, a recent network meta-analysis [45] reports that MT as a stand-alone treatment may be associated with greater improvements in pain levels than inert treatment in the short term but failed to find an association in the long-term follow-up. In the same study, NSAID medications were not more effective than inert treatment in the short-term follow up. No information was provided with regards to the long-term follow-up. A previous Cochrane systematic review [46] suggests that for acute and subacute neck pain, multiple sessions of MT were more effective than certain medications in improving pain at both short-term and long-term follow-up. However, the results of this review derived only from one trial. Our review identified an additional 8 trials, increasing confidence in the robustness of these results. With regards to adverse events, the relative literature presents conflicting results. Our findings are in agreement with a previous systematic review that reported that MT appears to have a relatively lower risk for adverse events compared to medications [47]. Previous evidence deriving from small observational studies and expert opinions has suggested a potential association between MT and craniocervical arterial dissections [48, 49]. Our analysis, however, does not confirm these findings. However, we found throughout our literature search that there was a paucity of reported adverse events for MT. Efficacy and safety of interventions in reporting is important for informing a policy perspective [20, 50]. Even though the included trials did not suggest any safety concerns, there was a lack of clarity around what events occurred, and to what percentage of the included participants adverse events were seen in. This is not uncommon in rehabilitation and medical literature [50, 51]. Recent literature suggests that reporting of adverse events within MT is low and does not always follow established standards [52, 53]. Conversely, when considering oral pain medication, trials and registries have developed processes for adverse event detection, processing, and reporting which has helped them to identify more adverse events [54]. Interestingly, when further examining our results, all-cause dropouts seemed to provide a higher quality estimate than the adverse events, and that could potentially be due to the higher number of events recorded. That further supports the possibility of inadequate reporting of adverse events, as certain individuals who dropped out without reason could have potentially experienced an adverse event that was not recorded.

### Strengths

We conducted an extended and comprehensive literature search across five databases and two trial registries with the help of an experienced librarian and we manually checked the reference lists of all the included studies for missed trials. We therefore believe that it’s unlikely that we missed any eligible trials. The robustness of our results is further enhanced by the low between trial heterogeneity in two out of four outcomes and the overall absence of publication bias. Our study included trials from Europe, North America, South America, Asia, and Australia and that further establishes the external validity and generalizability of our results.

### Limitations

The quality of our results is limited by the quality of the included studies. The certainty of evidence of our outcomes ranged from low to moderate. The most common reason for downgrading was high risk of bias, followed by inconsistency. High risk of bias is to be expected in trials of rehabilitation interventions, especially when compared with medications. The nature of the two interventions is very different, which makes effective blinding of the patients very challenging and can potentially introduce bias due to deviations from the intended interventions and, in cases of significant dropouts, bias due to missing outcome data. What is more, when the outcome is a patient reported outcomes such as pain, the patients act as the assessors, which introduces further bias in the measurement of the outcome.

Interestingly, the high level of heterogeneity that leads to inconsistency in pain intensity was only present in the short-term results, and not in the long-term ones. A potential reason that could explain this discrepancy is the large variability that is present in the short-term follow-up timepoint (ranging from 3 to 12 weeks), when compared to the long term follow up that was more standardized. There is also a possibility that the short-term timepoint was too soon for patients to accurately estimate the change in their pain levels, especially in the more chronic cases. This discrepancy could also be an indication of potentially more prolonged treatment effects in the patients that received MT compared to meds, that the short-term timepoint could not capture effectively.

Besides, a certain level of heterogeneity is also to be expected when pooling trials of MT interventions, mainly due to the complexity of the treatment, the large variability in treatment techniques, the potential between trials difference in the skill level of the therapist, and the multi-component nature of these interventions. MT interventions are rarely standardized. In most of the included trials, MT practitioners could choose from and utilize multiple options depending on the patient presentation, and the choice was usually left in expert opinion. What is more, certain MT techniques include mobilization/manipulation throughout the spine and not specifically to the neck, which could also have added to the heterogeneity. Other factors that could potentially explain the high level of heterogeneity are the familiarity, level of comfort and expectations of patients with regards to the MT techniques that might vary in different countries and that could also potentially explain the heterogeneity to some extend [15, 55, 56].

In order to facilitate clinical interpretation of our results, we used 0.4 SDs as the MID for pain, and 20% as the MID for adverse events. The current literature does not provide a between group MID for patients with neck pain for neither pain, nor adverse events. Although both 0.4SD for pain and 20% for adverse events can be realistic benchmarks within pain literature, we would like to acknowledge that they are not specific to neck pain patients.

### Clinical implications

The results of our study suggest that MT may be more effective for people with neck pain in both short-term, and in the long-term than oral pain medications. In the light of recent evidence that suggests the ineffectiveness of opioids for short-term pain in patients with non-specific neck pain [10] and taking into consideration the high risk of misuse that comes with long-term opioid use, our results could influence referral networks for individuals who present to their family physician with neck pain and give more treatment options for patients to choose from. According to our results, it appears that patients that receive MT may be at a lower risk for adverse events compared to patients receiving oral pain medications. The most common adverse even from medications according to a recent network-meta-analysis is gastric symptoms [45]. Hence, MT may be a good alternative to medications in patients with a history of gastrointestinal issues that may not be able to tolerate medications.

Finally, even though the results of our study do not suggest any safety concerns, taking into consideration the lack of adequate standardized reporting of the adverse events in MT, it is advised that clinicians that choose to use MT closely monitor for adverse events and follow up when patients discontinue the treatment program to further investigate the reasons behind it.

### Future research

Even though MT is a field that developed greatly in the past 10 years and is widely used in clinical practice, all but one of our studies were conducted more than 10 years ago. Hence, there is a possibility that there are new, emerging techniques in place that have not been tested against oral pain medications and are not included in our review. Future trials should focus on identifying and testing these techniques while integrating a strict reporting strategy for adverse evets to offer a wider choice of non-pharmacological therapeutic options to clinical practice.

## Conclusion

We found low certainty of evidence that MT interventions may be more effective than oral pain medication in pain reduction in the short-term, and moderate certainty of evidence that MT interventions may be more effective than oral pain medication in pain reduction in the long-term. We found low certainty evidence that the risk of adverse events may be lower for patients that received MT compared to the ones that received oral pain medication, but we advise caution when interpreting this result due to the different level of reporting strategies in place for MT and medication-induced adverse events. Future MT trials should create and adhere to strict reporting strategies with regards to adverse events to help gain a better understanding on the nature of potential MT-induced adverse events and to ensure patient safety.

### Electronic supplementary material

Below is the link to the electronic supplementary material.

Supplementary Material 1

Supplementary Material 2

## Data Availability

The datasets analyzed in this review are available from the corresponding author upon reasonable request.

## Abbreviations

MT: manual therapy
NSAIDs: nonsteroidal anti-inflammatory drugs
PRISMA: Preferred Reporting Items for Systematic Reviews and Meta-Analysis
CENTRAL: Cochrane Central Register of Controller Trials
AMED: Allied and Complementary Medicine
CINAHL: Cumulative Index to Nursing and Allied Health Literature
WAD: whiplash-associated disorder
AEs: adverse events
SAEs: serious adverse events
VAS: Visual Analogue Scale
RoB2: Cochrane Risk of Bias 2
GRADE: Grades of Recommendation, Assessment, Development, and Evaluation
SMDs: standardized mean differences
CI: confidence intervals
RRs: risk ratios
IQR: Interquartile Range
