# Supplementary material for: Effectiveness and safety of manual therapy when compared with oral pain medications in patients with neck pain: a systematic review and meta-analysis
Source: BMC Sports Sci Med Rehabil. 2024 Apr 16;16:86. doi: 10.1186/s13102-024-00874-w (PMC11020448; doi:10.1186/s13102-024-00874-w)
Supplement: Supplementary file 2 — Supplementary Material 2 [file 13102_2024_874_MOESM2_ESM.docx]

**Additional File**

**Effectiveness and Safety of Manual Therapy when Compared with Oral Pain Medications in Patients with Neck Pain: A Systematic Review and Meta-analysis.**

Joshua Makin*^1^, Lauren Watson*^1^, Dimitra V Pouliopoulou^2-3^, Taylor Laframboise^1^, Bradley Gangloff^1^, Ravinder Sidhu^1^, Jackie Sadi ^1^, Pulak Parikh^1^, Anita Gross^4^, Pierre Langevin^5-7^, Heather Gillis^1^, Pavlos Bobos^1-3^

*****Equally contributing authors

**Authors affiliations**

^1^Comprehensive Musculoskeletal Field, Advanced Health Care Program, School of Physical Therapy, Western University. London, ON, Canada.

^2^School of Physical Therapy, Health and Rehabilitation Sciences, Western University, London, Ontario, Canada

^3^Western’s Bone and Joint Institute, Collaborative Musculoskeletal Health Research Program, London, Ontario, Canada

^4^School of Rehabilitation Sciences, McMaster University

^5^School of Rehabilitation Sciences, Université Laval, Quebec City, Quebec, Canada

^6^School of Physical and Occupational Therapy, McGill University, Montréal, Canada

^7^Physio Interactive, Quebec City, Quebec, Canada

**Corresponding author**

Dr. Pavlos Bobos, School of Physical Therapy, Western University, 1201 Western Road, London ON, N6G 1H1, Elborn College, Rm 1443, Tel: 519-661-2111 ext. 86440, Email: [pbobos@uwo.ca](mailto:pbobos@uwo.ca)

Table of Contents

[Additional File 1*.* Research Strategy 3](#_Toc161404099)

[Additional File 2. Excluded studies - Reasons for exclusion 4](#_Toc161404100)

[Additional File 3. Interventions details 10](#_Toc161404101)

[Additional File 4. Summary of TiDiER Checklist 21](#_Toc161404102)

[Additional File 5.  Summary of the risk of bias assessment 22](#_Toc161404103)

[Additional File 6. Funnel Plot of short-term pain intensity 23](#_Toc161404104)

[Additional File 7. Funnel Plot of long-term pain intensity 24](#_Toc161404105)

[Additional File 8. GRADE evidence profile 25](#_Toc161404106)

[Additional File 9. Subgroup analysis for short-term pain intensity by treatment component 26](#_Toc161404107)

[Additional File 10. Subgroup analysis for long-term pain intensity by treatment component 27](#_Toc161404108)

#

# Additional File 1*.* Research Strategy

The Cochrane Central Register of Controlled Trials was searched through the Cochrane library (http://www.cochranelibrary.com/). The search was conducted on March 2023.

Cochrane Central Register of Controlled Trials (CENTRAL)

Search Name:

Date Run: 03/10/2022 18:44:14

Comment:

ID Search Hits

#1 ("Mulligan" OR "Maitland" OR "Kaltenborn" OR "manipulation" OR "mobilization" OR "Manual therapy"):ti,ab,kw

#2 MeSH descriptor: [Neck Pain] explode all trees

#3 (#1 AND #2) in Trials

# Additional File 2. Excluded studies - Reasons for exclusion

| **Study** | **Reasons for Exclusion** |
| --- | --- |
| Aboagye 2022(1) | Wrong comparator/Usual care – no medications |
| Afzal 2019(2) | Wrong comparator/Usual care – no medications |
| Alfawaz 2020(3) | Wrong comparator/Usual care – no medications |
| Arjona Retamal 2021(4) | Wrong comparator/Usual care – no medications |
| Ashfaq 2022(5) | Wrong comparator/Usual care – no medications |
| Ayub 2019(6) | Wrong comparator/Usual care – no medications |
| Barassi 2021(7) | Wrong comparator/Usual care – no medications |
| Basson 2014(8) | Wrong comparator/Usual care – no medications |
| Bautista – Aguirre 2017(9) | Wrong comparator/Usual care – no medications |
| Bevilaqua-Grossi 2016(10) | Wrong comparator/Usual care – no medications |
| Björklund 2012(11) | Wrong comparator/Usual care – no medications |
| Boyles 2010(12) | Wrong comparator/Usual care – no medications |
| Bronfort 2001(13) | Wrong comparator/Usual care – no medications |
| Calixtre 2019(14) | Wrong comparator/Usual care – no medications |
| Casanova- Méndez 2014(15) | Wrong comparator/Usual care – no medications |
| Cho 2019(16) | Wrong comparator/Usual care – no medications |
| Cleland 2005(17) | Wrong comparator/Usual care – no medications |
| Cleland 2010(18) | Wrong comparator/Usual care – no medications |
| Corum 2021(19) | Wrong comparator/Usual care – no medications |
| Cui 2017(20) | Wrong comparator/Usual care – no medications |
| De Camargo 2011(21) | Wrong comparator/Usual care – no medications |
| Desmoulin 2007(22) | Wrong intervention |
| Domingues 2019(23) | Wrong comparator/Usual care – no medications |
| Dunning 2016(24) | Wrong comparator/Usual care – no medications |
| Dziedzic 2005(25) | Wrong comparator/Usual care – no medications |
| Enthoven 2004(26) | Wrong study design (no RCT) |
| Erdem 2021(27) | Wrong comparator/Usual care – no medications |
| Evans 2012(28) | Wrong comparator/Usual care – no medications |
| Evans 2002(29) | Wrong comparator/Usual care – no medications |
| Evans 2003(30) | Wrong outcomes |
| Farooq 2018(31) | Wrong comparator/Usual care – no medications |
| Galindez-Ibarbengoetxea 2018(32) | Wrong comparator/Usual care – no medications |
| Galindez-Ibarbengoetxea 2018(33) | Wrong comparator/Usual care – no medications |
| García-Pérez-Juana 2018(34) | Wrong comparator/Usual care – no medications |
| Gattie 2021(35) | Wrong comparator/Usual care – no medications |
| Ghodrati 2020(36) | Wrong comparator/Usual care – no medications |
| Gilliani 2020(37) | Wrong comparator/Usual care – no medications |
| González Rueda 2017(38) | Wrong comparator/Usual care – no medications |
| González-Iglesias 2009(39) | Wrong comparator/Usual care – no medications |
| González-Rueda 2020(40) | Wrong comparator/Usual care – no medications |
| González-Rueda 2021(41) | Wrong comparator/Usual care – no medications |
| Griswold 2018(42) | Wrong comparator/Usual care – no medications |
| Groeneweg 2010(43) | Wrong comparator/Usual care – no medications |
| Groeneweg 2017(44) | Wrong study design (no RCT) |
| Groisman 2020(45) | Wrong comparator/Usual care – no medications |
| Gudavalli 2015(46) | Wrong comparator/Usual care – no medications |
| Haas 2003(47) | Wrong comparator/Usual care – no medications |
| Haas 2010(48) | Wrong comparator/Usual care – no medications |
| Haas 2004(49) | Wrong comparator/Usual care – no medications |
| Haavik-Taylor 2007(50) | Wrong study design (no RCT) |
| Haleema 2021(51) | Wrong comparator/Usual care – no medications |
| Hall 2007(52) | Wrong comparator/Usual care – no medications |
| Hanney 2017(53) | Wrong patient population |
| Harrison 2003(54) | Wrong study design (no RCT) |
| Harrison 2004(55) | Wrong study design (no RCT) |
| Heikkilä 2000(56) | Wrong outcomes |
| Holm 2021(57) | Wrong comparator/Usual care – no medications |
| Jensen 2009(58) | Wrong study design (no RCT) |
| Jordan 1998(59) | Wrong comparator/Usual care – no medications |
| Kanlayanaphotporn 2010(60) | Wrong comparator/Usual care – no medications |
| Kim 2022(61) | Wrong intervention |
| Kim 2017(62) | Wrong comparator/Usual care – no medications |
| Klein 2013(63) | Wrong comparator/Usual care – no medications |
| Kongsted 2007(64) | Wrong comparator/Usual care – no medications |
| Korthals-de Bos 2003(65) | Wrong comparator/Usual care – no medications |
| Langenfeld 2015(66) | Wrong comparator/Usual care – no medications |
| Langevin 2012(67) | Wrong comparator/Usual care – no medications |
| Lascurain-Aguirrebeña 2021(68) | Wrong comparator/Usual care – no medications |
| Leininger 2016(69) | Wrong comparator/Usual care – no medications |
| Leininger 2014(70) | Wrong study design (no RCT) |
| Lewis 2007(71) | Wrong comparator/Usual care – no medications |
| Licht 1998(72) | Wrong comparator/Usual care – no medications |
| Lin 2013(73) | Wrong comparator/Usual care – no medications |
| Lizis 2020(74) | Wrong comparator/Usual care – no medications |
| Lohman 2019(75) | Wrong comparator/Usual care – no medications |
| López-de-Uralde-Villanueva 2020(76) | Wrong comparator/Usual care – no medications |
| Lopez-Lopez 2015(77) | Wrong comparator/Usual care – no medications |
| Mahmood 2021(78) | Wrong comparator/Usual care – no medications |
| Maiers 2014(79) | Wrong comparator/Usual care – no medications |
| Maiers 2019(80) | Wrong comparator/Usual care – no medications |
| Maiers 2007(81) | Protocol (no RCT results) |
| Mansilla-Ferragut 2009(82) | Wrong comparator/Usual care – no medications |
| Martínez-Segura 2012(83) | Wrong comparator/Usual care – no medications |
| Masaracchio 2013(84) | Wrong comparator/Usual care – no medications |
| McDevitt 2022(85) | Wrong comparator/Usual care – no medications |
| McReynolds 2005(86) | Wrong intervention |
| Moser 2019(87) | Wrong comparator/Usual care – no medications |
| Moulson 2006(88) | Wrong patient population |
| Moustafa 2017(89) | Wrong comparator/Usual care – no medications |
| Murphy 2010(90) | Wrong comparator/Usual care – no medications |
| Nasir 2021(91) | Wrong comparator/Usual care – no medications |
| NCT00030004 2002(92) | Protocol (no RCT results) – was terminated |
| NCT00269360 2005(93) | Protocol (no RCT results) |
| NCT00416117 2006(94) | Protocol (no RCT results) |
| NCT00429624 2007(95) | Protocol (no RCT results) |
| NCT00713843 2008(96) | Wrong comparator/usual care – no medications |
| NCT00978094 2009 (97) | Wrong comparator/Usual care – no medications |
| NCT01161758 2010(98) | Wrong comparator/Usual care - no medications |
| NCT01318720 2011(99) | Wrong comparator/Usual care – no medications |
| NCT01745705 2012(100) | Wrong comparator/Usual care – no medications |
| NCT01938209 2013(101) | Wrong comparator/Usual care – no medications |
| NCT01962090 2013(102) | Wrong comparator/Usual care – no medications |
| NCT02036905 2013(103) | Wrong comparator/Usual care – no medications |
| NCT02051478 2014(104) | Wrong comparator/Usual care – no medications |
| NCT02245425 2014(105) | Wrong comparator/Usual care – no medications |
| NCT02287337 2014(106) | Wrong comparator/Usual care – no medications |
| NCT02301871 2014(107) | Wrong intervention |
| NCT02356380 2015(108) | Wrong study design (no RCT) |
| NCT02415660 2015(109) | Wrong comparator/Usual care – no medications |
| NCT02435966 2015(110) | Wrong comparator/Usual care – no medications |
| NCT02552290 2015(111) | Wrong comparator/Usual care – no medications |
| NCT02645630 2015(112) | Wrong comparator/Usual care – no medications |
| NCT02667821 2016(113) | Wrong comparator/Usual care – no medications |
| NCT02691143 2016(114) | Wrong comparator/Usual care – no medications |
| NCT02731014 2016(115) | Protocol (no RCT results) |
| NCT02772042 2016(116) | Wrong comparator/Usual care – no medications |
| NCT02832232 2016(117) | Wrong comparator/Usual care – no medications |
| NCT02927977 2016(118) | Wrong comparator/Usual care – no medications |
| NCT02972710 2016(119) | Wrong comparator/Usual care – no medications |
| NCT02982369 2016(120) | Wrong comparator/Usual care – no medications |
| NCT03012503 2017(121) | Wrong comparator/Usual care – no medications |
| NCT03149302 2017(122) | Wrong comparator/Usual care – no medications |
| NCT03157349 2017(123) | Wrong intervention |
| NCT03176654 2017(124) | Wrong comparator/Usual care – no medications |
| NCT03187808 2017(125) | Wrong intervention |
| NCT03190187 2017(126) | Wrong comparator/Usual care – no medications |
| NCT03194490 2017(127) | Wrong comparator/Usual care – no medications |
| NCT03294785 2017(128) | Protocol (no RCT results) |
| NCT03385889 2017(129) | Wrong comparator/Usual care – no medications |
| NCT03447977 2017(130) | Wrong comparator/Usual care – no medications |
| NCT03453203 2018(131) | Wrong comparator/Usual care – no medications |
| NCT03507907 2018(132) | Wrong comparator/Usual care – no medications |
| NCT03509649 2018(133) | Wrong comparator/Usual care – no medications |
| NCT03560947 2018(134) | Wrong comparator/Usual care – no medications |
| NCT03562338 2018(135) | Wrong comparator/Usual care – no medications |
| NCT03563079 2018(136) | Wrong comparator/Usual care – no medications |
| NCT03903380 2019(137) | Wrong comparator/Usual care – no medications |
| NCT04054869 2019 (138) | Wrong comparator/Usual care – no medications |
| NCT04182035 2019(139) | Wrong comparator/Usual care – no medications |
| NCT04268667 2020(140) | Wrong comparator/Usual care – no medications |
| NCT04306640 2020(141) | Wrong comparator/Usual care – no medications |
| NCT04327739 2020(142) | Wrong comparator/Usual care – no medications |
| NCT04351971 2020(143) | Wrong intervention |
| NCT04440293 2020(144) | Wrong intervention |
| NCT04455048 2020(145) | Wrong comparator/Usual care – no medications |
| NCT04545996 2020(146) | Wrong comparator/Usual care – no medications |
| NCT04556955 2020(147) | Wrong comparator/Usual care – no medications |
| NCT04610255 2020(148) | Wrong comparator/Usual care – no medications |
| NCT04660292 2020(149) | Protocol (no RCT results) |
| NCT04702100 2021(150) | Wrong comparator/Usual care – no medications |
| NCT04768790 2021(151) | Wrong comparator/Usual care – no medications |
| NCT04777890 2021(152) | Wrong comparator/Usual care – no medications |
| NCT04813315 2021(153) | Wrong comparator/Usual care – no medications |
| NCT04856813 2021(154) | Wrong comparator/Usual care – no medications |
| NCT04924764 2021(155) | Wrong study design (no RCT) |
| NCT04930575 2021(156) | Wrong comparator/Usual care – no medications |
| NCT05004467 2021(157) | Wrong comparator/Usual care – no medications |
| NCT05098860 2021(158) | Wrong comparator/Usual care – no medications |
| NCT05125250 2021(159) | Wrong intervention |
| NCT05186584 2022(160) | Wrong comparator/Usual care – no medications |
| NCT05226559 2022(161) | Protocol (no RCT results) |
| NCT05227963 2022(162) | Wrong comparator/Usual care – no medications |
| NCT05257616 2022(163) | Wrong comparator/Usual care – no medications |
| NCT05272111 2022(164) | Wrong comparator/Usual care – no medications |
| NCT05308199 2022(165) | Wrong comparator/Usual care – no medications |
| NCT05315076 2022(166) | Protocol (no RCT results) |
| NCT05350254 2022(167) | Protocol (no RCT results) |
| NCT05374057 2022(168) | Protocol (no RCT results) |
| NCT05391997 2022(169) | Wrong intervention |
| NCT05392465 2022(170) | Protocol (no RCT results) |
| NCT05399953 2022(171) | Protocol (no RCT results) |
| NCT05404659 2022(172) | Wrong comparator/Usual care – no medications |
| NCT05410067 2022(173) | Wrong comparator/Usual care – no medications |
| NCT05425706 2022(174) | Wrong comparator/Usual care – no medications |
| NCT05474456 2022(175) | Wrong study design (no RCT) |
| NCT05496699 2022(176) | Wrong intervention |
| NCT05502406 2022(177) | Wrong comparator/Usual care – no medications |
| Nee 2013(178) | Wrong study design (no RCT) |
| Nee 2012(179) | Wrong comparator/Usual care – no medications |
| Ogura 2011(180) | Wrong comparator/Usual care – no medications |
| Paanalahti 2014(181) | Wrong comparator/Usual care – no medications |
| Palmgren 2006(182) | Wrong comparator/Usual care – no medications |
| Park 2021(183) | Wrong comparator/Usual care – no medications |
| Peña-Salinas 2017(184) | Wrong comparator/Usual care – no medications |
| Pillastrini 2016(185) | Wrong comparator/Usual care – no medications |
| Pillastrini 2018(186) | Wrong comparator/Usual care – no medications |
| Pires 2015(187) | Wrong comparator/Usual care – no medications |
| Plaza-Manzano 2014(188) | Wrong comparator/Usual care – no medications |
| Pool 2006(189) | Wrong comparator/Usual care – no medications |
| Puentedura 2011(190) | Wrong comparator/Usual care – no medications |
| Puerma-Castillo 2018(191) | Wrong comparator/Usual care – no medications |
| Rampazo 2021(192) | Wrong comparator/Usual care – no medications |
| Razzaq 2020(193) | Wrong comparator/Usual care – no medications |
| Reid 2012(194) | Wrong comparator/Usual care – no medications |
| Reid 2008(195) | Wrong comparator/Usual care – no medications |
| Rodríguez-Sanz 2017(196) | Wrong comparator/Usual care – no medications |
| Rogers 1997(197) | Wrong study design (no RCT) |
| Romero Del Rey 2022(198) | Wrong comparator/Usual care – no medications |
| Rosenfeld 2003(199) | Wrong comparator/Usual care – no medications |
| Ruiz-Sáez 2007(200) | Wrong patient population |
| Saavedra-Hernández 2012(201) | Wrong comparator/Usual care – no medications |
| Saavedra-Hernández 2013(202) | Wrong comparator/Usual care – no medications |
| Saayman 2011(203) | Wrong comparator/Usual care – no medications |
| Savva 2021(204) | Wrong comparator/Usual care – no medications |
| Schwerla 2008(205) | Wrong comparator/Usual care – no medications |
| Siddiqui 2022(206) | Wrong comparator/Usual care – no medications |
| Skargren 1997(207) | Wrong comparator/Usual care – no medications |
| Skillgate 2007(208) | Wrong comparator/Usual care – no medications |
| Skillgate 2010(209) | Wrong comparator/Usual care – no medications |
| Sparks 2017(210) | Wrong comparator/Usual care – no medications |
| Sremakaew 2018(211) | Wrong comparator/Usual care – no medications |
| Stieven 2020(212) | Wrong intervention |
| Van Schalkwyk 2000(213) | Wrong comparator/Usual care – no medications |
| Vernon 2012(214) | Wrong comparator/Usual care – no medications |
| Vernon 2013(215) | Wrong comparator/Usual care – no medications |
| Von Piekartz 2013(216) | Wrong comparator/Usual care – no medications |
| Walker 2013(217) | Wrong comparator/Usual care – no medications |
| Williams 2003(218) | Wrong comparator/Usual care – no medications |
| Williams 2004(219) | Wrong comparator/Usual care – no medications |
| Wood 2001(220) | Wrong comparator/Usual care – no medications |
| Yang 2014(221) | Wrong comparator/Usual care – no medications |
| Young 2019(222) | Wrong comparator/Usual care – no medications |
| Yung 2020(223) | Wrong comparator/Usual care – no medications |
| Zaproudina 2007(224) | Wrong comparator/Usual care – no medications |

# Additional File 3. Interventions details

Bronfort, 2012. Description of intervention based on TiDiER checklist

| Intervention Name (brief) | Spinal manipulation vs medication vs exercise |
| --- | --- |
| Why (rationale of treatment) | Neck pain is a clinically relevant aspect of medicine, with many patients seeking care on an annual basis. There is a large healthcare burden with managing neck pain. Having a better understanding of the effectiveness of varying interventions on neck pain can help improve outcomes for patients. |
| What | Patients were divided randomly into 3 groups: spinal manipulative therapy, medication and home exercise with advice. The spinal manipulation group received HVLA and LVLA techniques to affected segments over the course of 12 weeks with each session lasting 20-30 minutes. The medication group was provided medication prescriptions beginning with NSAIDS and Acetaminophen then progressing to narcotics, if required, along with advice to stay active. The home exercise and advice groups were administered in 2- 1 hour in person sessions with a home booklet of exercises to complete. Provided by booklet |
| Where: (infrastructure and relevant features) | The trial was conducted in a university affiliated outpatient clinic and a pain management clinic in Minnesota. |
| How: (modes of delivery) | Who: SMT group was administered by 6 chiropractors with >5 years of experience. Medications were prescribed by a licensed medical physician. Home exercises were taught by 6 therapists |
| When and how much: (number of sessions, duration, intensity or dose) | When and how much: SMT- 12 weeks of 20–30-minute sessions, medication prescription was scheduled based on patient history and consisted of 15–20 minute visits, HEA program consisted of 2-1 hours sessions separated by 1-2 weeks. |
| Tailoring (personalization) | The SMT and medication groups had sessions scheduled based on the recommendations of the providing chiropractor/physician based on the individual needs of the patient. |
| Modifications (from existing or initial protocol) | N/A |
| How well: planned (adherence and procedure to maintain it) | Participants were monitored at 2,4,6,8 and 12 week follow ups, with data collected at each time point. Post intervention follow up was collected at 26 weeks and 52 weeks.  We first imputed values to the missing responses of these 12 participants by using the mean percentage reduction from baseline at all time points specific to the group to which they belonged. Then, we imputed the rest of the missing data during treatment and the 2 posttreatment follow-up time points by using the SAS multiple imputation strategy, on the assumption that the data were missing at random. |

Calvo-Lobo 2018. Description of intervention based on TiDiER checklist

| Intervention Name (brief) | Pharmacological Vs Neural mobilizations for Cervicobrachial pain |
| --- | --- |
| Why (rationale of treatment) | Cervicobrachial pain has an estimated incidence of 83 in100,000 (Calvo-Lobo 2018), with approximately 19.9 % estimated to be neuropathic in origin with nerve trunk mechanosentivity. Specific therapies targeting CP are believed to assist in recovery from CB. |
| What | Patients were screened for CB and randomly assigned to 1 of 3 groups:  Group 1: Median nerve neurodynamic mobilization group for 5 interventions per week (daily Monday to Friday) with neurodynamic mobilizations described by Butler in article  Group 2: Pharmacological treatment of oral ibuprofen into 3 doses per day  Group 3: Neural mobilization using a contralateral gliding technique for 5 intervention sessions per week (Monday to Friday) |
| Where: (infrastructure and relevant features) | The study was conducted in Venezuela |
| How: (modes of delivery) | In person intervention was administered by a physiotherapist and physician on an individual basis |
| When and how much: (number of sessions, duration, intensity or dose) | Group 1 and 3 consisted of 5 sessions per week for 6 weeks, group 2 session frequency was not reported |
| Tailoring (personalization) | N/A |
| Modifications (from existing or initial protocol) | This study was a combination of 2 separate studies with a deviation from sample size calculations to include 3 cohorts. |
| How well: planned (adherence and procedure to maintain it) | There was no planned or actual intervention monitoring or assessment. |

De Hertogh 2009. Description of intervention based on TiDiER checklist

| Intervention Name (brief) | Manual therapy and usual care |
| --- | --- |
| Why (rationale of treatment) | Headaches are a common disorder in which physical treatments are often utilized. There is insufficient evidence regarding the value of these treatments in medical care |
| What | Patients were provided with a letter containing general recommendations for treatment and available evidence for the UCMT group. The UC group followed the Dutch college of GP’s protocol.  UCMT group received a combination of spinal mobilizations and low-load stabilizing exercise therapy |
| Where: (infrastructure and relevant features) | Patients were recruited at GP offices and university hospitals in UZ Brussel and UZA, no information was provided for intervention locations  How: in person delivery by therapist |
| Who: | The interventions were administered by “therapist”, without mention of designation |
| How: (modes of delivery) | In person sessions |
| When and how much: (number of sessions, duration, intensity or dose) | UCMT group received a maximum of 12 sessions (2x/wk. over 6 weeks) |
| Tailoring (personalization) | Individualized interventions as decided by therapist, as well as individualized care by GP |
| Modifications (from existing or initial protocol) | N/A |
| How well: planned (adherence and procedure to maintain it) | Adherence was monitored with reasoning for drop out being reported in many cases. It is not clear how the adherence was evaluated; however, all patients were accounted for |

Giles, 1999. Description of intervention based on TiDiER checklist

| Intervention Name (brief) | Spinal manipulation, acupuncture |
| --- | --- |
| Why (rationale of treatment) | There is a large healthcare burden from chronic spinal related pain, with limited knowledge of the best treatment modality. |
| What | - 1. Acupuncture performed for 20 minutes using disposable 0.25mm gauge x 50mm length needles with low volt electrical stimulation   2. Spinal manipulation was performed as deemed by chiropractor   3. Non steroid anti-inflammatory medications were provided in a 3–4-week treatment period |
| Where: (infrastructure and relevant features) | Townsville General hospital outpatient spinal pain unit, Australia |
| How: (modes of delivery) | In person visits |
| When and how much: (number of sessions, duration, intensity or dose) | 6 treatments over a 3–4-week period standardized to 15–20-minute sessions |
| Tailoring (personalization) | Individual acupuncture points were used based on presentation, unclear if individualization of spinal manipulation or NSAIDS were used |
| Modifications (from existing or initial protocol) | Inadequate staffing led to halting randomization before all envelopes were used, altered timelines for intervention and poor acupuncture scheduling due to busy schedules |
| How well: planned (adherence and procedure to maintain it) | Monitoring was conducted with drop out follow up completed via telephone, with reasons for drop out noted |

Hoving 2002. Description of intervention based on TiDiER checklist

| Intervention Name (brief) | Manual Therapy |
| --- | --- |
| Why (rationale of treatment) | Neck pain is a common problem and can be severely disabling and costly. It is known that manual therapy is a part of multimodal conservative treatment of neck pain; however, further detailed studies are required. The techniques in the context of this article include muscular mobilizations, specific articular mobilizations, coordination, and stabilization techniques. These techniques can improve soft tissue function, improve joint function and decrease restrictions in movement at single/multiple segmental levels, and improve postural control, coordination, and movement patterns by using stabilizing cervical musculature, respectively. |
| What | Manual therapy included muscular mobilizations, specific articular mobilizations, coordination and stabilization techniques at the discretion of the therapist |
| Where: (infrastructure and relevant features) | 4 different research centers in the Netherlands |
| How: (modes of delivery) | Required experienced manual therapists acknowledged by the Netherlands Manual Therapy Association |
| When and how much: (number of sessions, duration, intensity or dose) | The intervention period lasted 6 weeks where patients were allowed to perform exercises at home and continue medication at baseline or over the counter use, other co-interventions were discouraged but were registered if they occurred, intensity/dose at the discretion of the therapist    Forty-five-minute treatment sessions were scheduled once per week, for a maximum of six treatments. A maximum number of visits was set for each intervention group; however, the patients did not have to complete this maximum number if symptoms had resolved. |
| Tailoring (personalization) | Within the boundaries of the protocol, treatment could be reassessed and adapted to the patient’s condition. The specific treatment characteristics were registered at each visit. |
| Modifications (from existing or initial protocol) | Manual therapy was up to the discretion of the therapist but no apparent deviations from protocol. One participant deviated from protocol by receiving Mensendieck exercise therapy and 5 participants had GP consultations during the initial 6 weeks |
| How well: planned (adherence and procedure to maintain it) | Patients were to register any deviations from the protocol, other than this reporting and discouraged patients from cointerventions the article does not appear to have any specific planned adherence procedures |

Hoving 2006. Description of intervention based on TiDiER checklist

| Intervention Name (brief) | Manual Therapy |
| --- | --- |
| Why (rationale of treatment) | Neck pain is a common and painful MSK condition. It is known that manual therapy can be a component of treatment of this condition; however, few RCTs determining the effectiveness of conservative treatments |
| What | Manual therapy included muscular mobilizations, specific articular mobilizations, coordination, and stabilization techniques at the discretion of the therapist |
| Where: (infrastructure and relevant features) | 4 different research centers in the Netherlands |
| How: (modes of delivery) | Required experienced manual therapists acknowledged by the Netherlands Manual Therapy Association |
| When and how much: (number of sessions, duration, intensity or dose) | The intervention period lasted 6 weeks where patients were allowed to perform exercises at home and continue medication at baseline or over the counter use, other co-interventions were discouraged but were registered if they occurred, intensity/dose at the discretion of the therapist    45-minute treatment sessions were scheduled once per week, for a maximum of six treatments. A maximum number of visits was set for each intervention group; however, the patients did not have to complete this maximum number if symptoms had resolved. |
| Tailoring (personalization) | Within the boundaries of the protocol, treatment could be reassessed and adapted to the patient’s condition. The specific treatment characteristics were registered at each visit.  After the 6-week period any further treatment was up to the discretion of the patient’s GP. |
| Modifications (from existing or initial protocol) | Manual therapy was up to the discretion of the therapist. No apparent modification/deviations from protocol other than patients receiving additional treatments, which was reported in Table 2. |
| How well: planned (adherence and procedure to maintain it) | Patients were to register any deviations from the protocol. Other than encouraging patients to report other treatments (which is reported in Table 2) and discouraging co-interventions, the article does not appear to have any specific planned adherence procedures |

Lee, 2021. Description of intervention based on TiDiER checklist

| Intervention Name (brief) | Chuna Manual therapy |
| --- | --- |
| Why (rationale of treatment) | Neck pain is the second most prevalent musculoskeletal condition globally, with a high socioeconomic burden. Multiple treatment modalities have been examined including conventional and alternative medicine practices. |
| What | There were no physical materials provided to the patients. Both groups were provided 10 sessions of Chuna manual therapy or usual care consisting of medications and electrotherapy |
| Where: (infrastructure and relevant features) | Hospitals designated as spinal specialty hospitals by the Korean Ministry of Health and Welfare (Jaseng Hospital of Korean Medicine at Gangnam, Bucheon, Daejeon, and Haeundae) and 1 academic Korean medicine hospital (Kyung) Hee University Korean Medicine Hospital at Gangdong). |
| How: (modes of delivery) | The program was administered by 3 Korean medicine physicians with minimum 3 years of experience in Chuna medicine |
| When and how much: (number of sessions, duration, intensity or dose) | Each group completed 10 sessions at a frequency of 2 sessions per week for 5 weeks. |
| Tailoring (personalization) | Chuna techniques and medication prescription were selected based on the recommendations of the treating physician |
| How well: planned (adherence and procedure to maintain it) | Participants were monitored on a quarterly basis for 1 year with no mention of the communication method.  The authors commented on monitoring patients who withdrew from the study; however, the authors did not comment on the reasoning for the withdrawal or the method for follow up. |

Muller 2005. Description of intervention based on TiDiER checklist

| Intervention Name (brief) | Spinal manipulation, acupuncture |
| --- | --- |
| Why (rationale of treatment) | Mechanical spinal pain is often a challenging condition to diagnose and treat. The search for an effective conservative technique has led to inconclusive results |
| What | Medication patients were normally given celecoxib (Celebrex) (200 to 400 mg/d; 27 patients) unless celecoxib had previously been tried; the next drug of choice was rofecoxib (Vioxx) (12.5 to 25 mg/d; 11 patients), followed by acetaminophen (paracetamol) (500-mg tablets 2-6 per day; 5 patients). Acupuncture was performed using sterile HWATO Chinese Acupuncture Guide Tube Needles (50 mm long; 0.25-mm gauge) for 20-minute appointments. For each patient, 8 to 10 needles were placed in local paraspinal intramuscular pain areas, and approximately 5 needles were placed in distal acupuncture point meridians according to the “Near and Far” technique (upper limb, lower limb, or scalp). High-velocity low amplitude spinal manipulative thrust to a joint was performed as judged safe and usual treatment by the treating chiropractor for the spinal level of involvement to mobilize the spinal joints at that level. |
| Where: (infrastructure and relevant features) | Multidisciplinary spinal pain unit of Townsville’s General Hospital, Australia |
| Who: | The medication intervention was administered by a physician, the administration of acupuncture and high velocity group was not clearly presented |
| How: (modes of delivery) | In person |
| When and how much: (number of sessions, duration, intensity or dose) | The acupuncture and high velocity group were treated with 2 twenty-minute sessions per week, the medication group was followed “fortnightly” for 20-minute sessions. All treatment modalities were followed until the patient was asymptomatic |
| Tailoring (personalization) | The medication group had a choice of medications based on their needs and was able to be tailored based on the individual medication history. |
| How well: planned (adherence and procedure to maintain it) | Follow up was completed via questionnaire and was submitted between 12 months and 36 months post intervention.  The authors monitored for drop out and co-intervention status. |

Walker, 2008. Description of intervention based on TiDiER checklist

| Intervention Name (brief) | Manual therapy and exercise |
| --- | --- |
| Why (rationale of treatment) | There is evidence that the use of manual therapy and exercise can be beneficial for mechanical type neck pain. This study’s aim was to help identify its effectiveness |
| What | Patients in the manual therapy/exercise group received physiotherapy targeted at impairments based on the physical examination, with a standardized exercise program provided (along with additional exercises if deemed relevant by the therapist). Patients in the minimal intervention group received posture education, encouragement of activities of daily living and continued medication use if appropriate. The minimal intervention group also received a 10-minute ultrasound treatment consisting of 10% pulse at 0.1w/cm^2^. |
| Where: (infrastructure and relevant features) | Military treatment centers |
| Who: | Physical therapists |
| How: (modes of delivery) | In person |
| When and how much: (number of sessions, duration, intensity or dose) | Both intervention periods were 3 weeks with both groups receiving 6 total treatments at a frequency of 2 sessions per week |
| Tailoring (personalization) | The treating physical therapist had autonomy to prescribe additional exercises and a choice in the type of manual technique provided to patient |
| How well: planned (adherence and procedure to maintain it) | The follow up was completed at 3, 6 weeks and 1 year post intervention.  It was not clear how the participants were followed or the reasons for any dropouts. |

Giles, 2003. Description of intervention based on TiDiER checklist

| Intervention Name (brief) | Spinal manipulation, acupuncture, medication |
| --- | --- |
| Why (rationale of treatment) | There is an uncertainty around the value of varying modalities for treating chronic spinal pain. This results in significant health care spending. |
| What | Acupuncture performed for 20 minutes using disposable 0.25mm gauge x 50mm length needles, using the Near and Far technique.  Spinal manipulation was performed as deemed by chiropractor in a 20-minute session.  Non steroid anti-inflammatory medications were provided in a 20-minute appointment |
| Where: (infrastructure and relevant features) | Townsville General hospital outpatient spinal pain unit, Australia |
| How: (modes of delivery) | In person visits |
| When and how much: (number of sessions, duration, intensity or dose) | 2 treatments per week for up to 9 weeks, early recovery defined at 2 week or 5 week point |
| Tailoring (personalization) | Individual acupuncture points were used based on presentation, individualized spinal manipulation was selected.  Medication was individualized based on patient assessment and previous medications (Celebrex, Vioxx followed by Paracetamol) |
| Modifications (from existing or initial protocol) | No modifications |
| How well: planned (adherence and procedure to maintain it) | Monitoring was conducted with drop out - reasons for drop out were noted |

# Additional File 4. Summary of TiDiER Checklist

| TiDiER checklist item | | | | | | | | | | | | |
| --- | --- | --- | --- | --- | --- | --- | --- | --- | --- | --- | --- | --- |
| Author | 1 | 2 | 3 | 4 | 5 | 6 | 7 | 8 | 9 | 10 | 11 | 12 |
| Brontfort 2012 | 🗸 | 🗸 | 🗸 | 🗸 | 🗸 | 🗸 | 🗸 | 🗸 | 🗸 | N/A | 🗸 | 🗸 |
| Calvo-Lobo 2018 | 🗸 | 🗸 | X | 🗸 | 🗸 | 🗸 | 🗸 | 🗸 | N/A | 🗸 | N/A | N/A |
| De Hertogh 2009 | 🗸 | 🗸 | 🗸 | 🗸 | 🗸 | 🗸 | 🗸 | 🗸 | 🗸 | N/A | 🗸 | 🗸 |
| Giles 1999 | 🗸 | 🗸 | X | 🗸 | 🗸 | 🗸 | 🗸 | 🗸 | 🗸 | 🗸 | 🗸 | 🗸 |
| Hoving 2002 | 🗸 | 🗸 | X | 🗸 | 🗸 | 🗸 | 🗸 | 🗸 | 🗸 | 🗸 | X | X |
| Hoving 2006 | 🗸 | 🗸 | X | 🗸 | 🗸 | 🗸 | 🗸 | 🗸 | 🗸 | 🗸 | X | X |
| Lee 2021 | 🗸 | 🗸 | X | 🗸 | 🗸 | 🗸 | 🗸 | 🗸 | 🗸 | X | 🗸 | 🗸 |
| Muller 2005 | 🗸 | 🗸 | X | 🗸 | 🗸 | 🗸 | 🗸 | 🗸 | 🗸 | X | 🗸 | 🗸 |
| Walker 2008 | 🗸 | 🗸 | 🗸 | 🗸 | 🗸 | 🗸 | 🗸 | 🗸 | 🗸 | X | 🗸 | X |
| Giles 2003 | 🗸 | 🗸 | X | 🗸 | 🗸 | 🗸 | 🗸 | 🗸 | 🗸 | X | 🗸 | 🗸 |

1. Brief name

2. Why (rationale, theory, goal)

3. Materials used in intervention (physical or informational)

4. Procedures (activities, processes)

5. Intervention providers

6. Modes of delivery (e.g. face-to-face, virtual, individual or group)

7. Location of intervention

8. When and how much (number of sessions, time period, schedule and dose)

9. Details of tailoring

10. Modifications to intervention

11. Planned adherence and fidelity assessment (key words used: adherence, compliance, fidelity, engagement, participation, commitment)

12. Actual adherence and fidelity (key words used: adherence, compliance, fidelity, engagement, participation, commitment)

# Additional File 5.  Summary of the risk of bias assessment


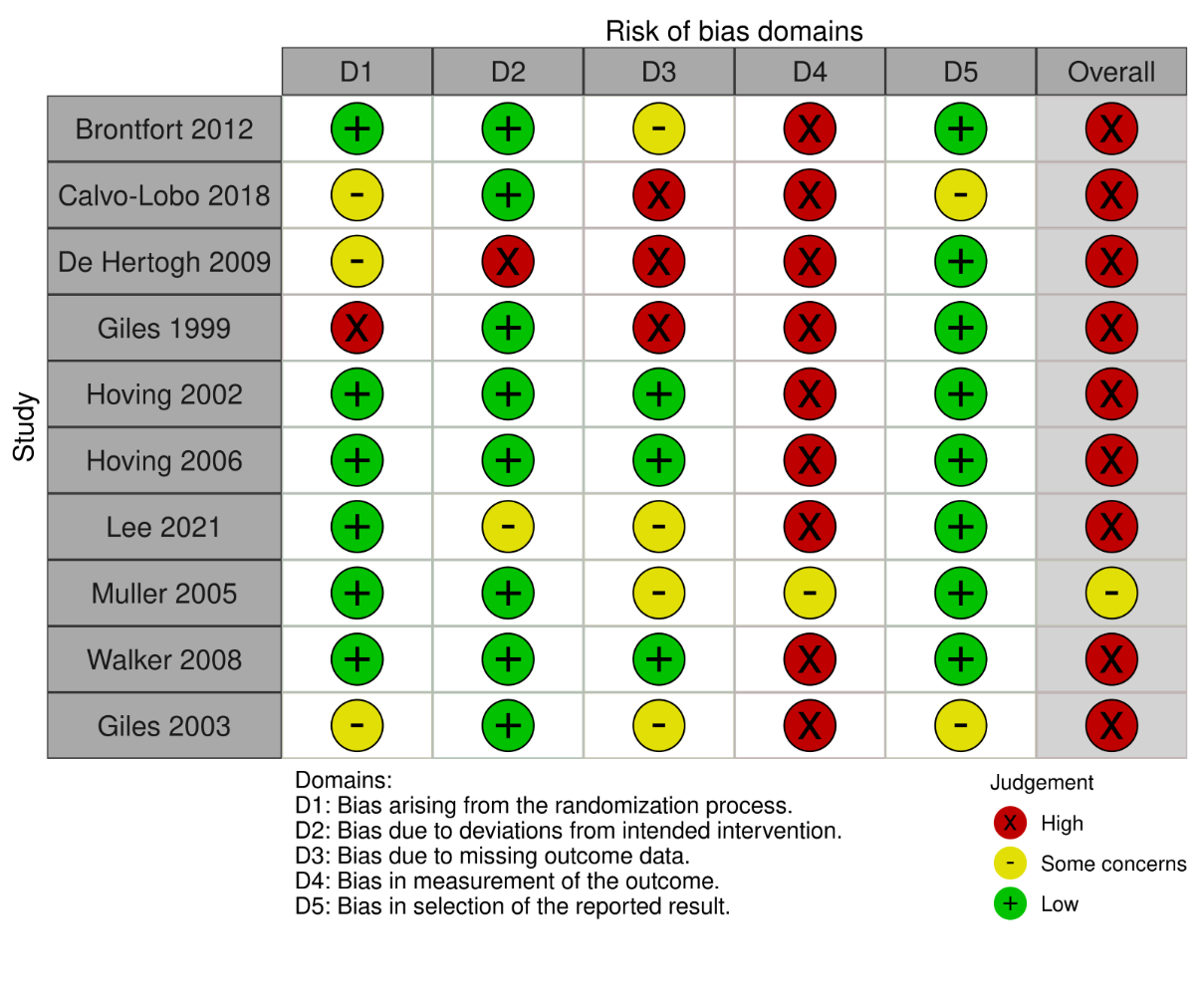


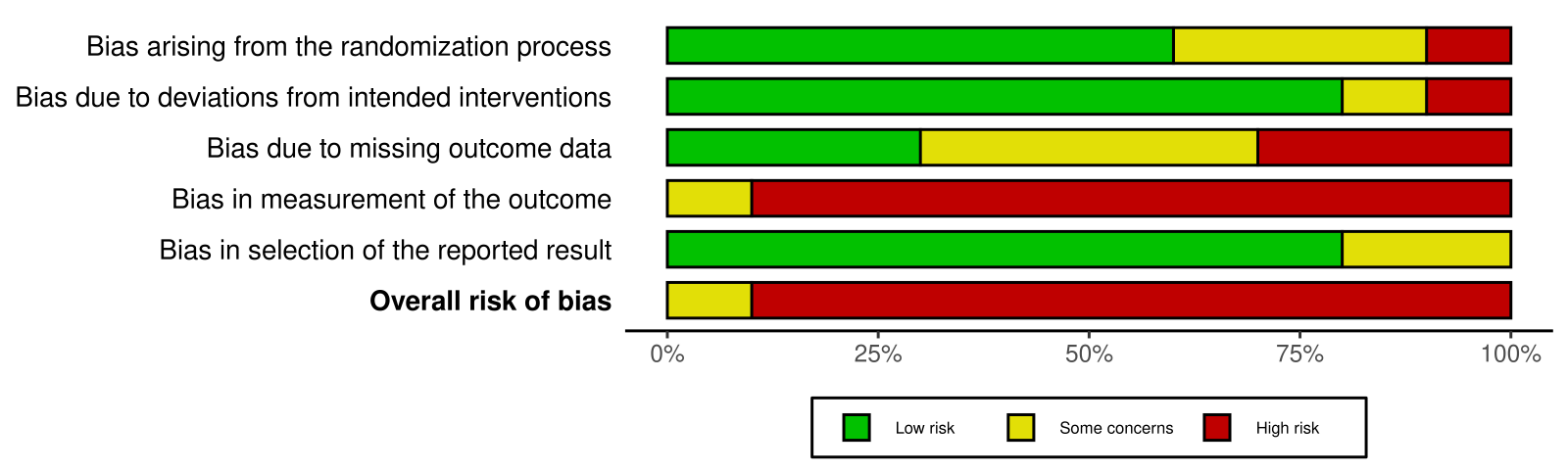


McGuinness, LA, Higgins, JPT. Risk-of-bias VISualization (robvis): An R package and Shiny web app for visualizing risk-of-bias assessments. Res Syn Meth. 2020; 1- 7. <https://doi.org/10.1002/jrsm.1411>

# Additional File 6. Funnel Plot of short-term pain intensity


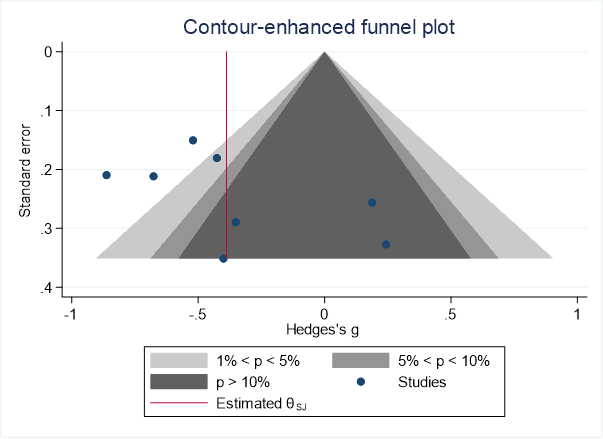


Regression-based Egger test for small-study effects

Random-effects model

Method: Sidik-Jonkman

H0: beta1 = 0; no small-study effects

beta1 = 2.85

SE of beta1 = 2.073

z = 1.38

Prob > |z| = 0.1688

# Additional File 7. Funnel Plot of long-term pain intensity


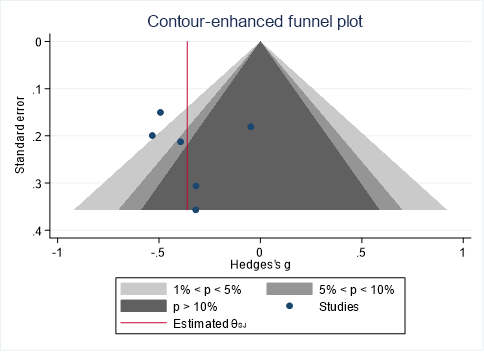


Regression-based Egger test for small-study effects

Random-effects model

Method: Sidik-Jonkman

H0: beta1 = 0; no small-study effects

beta1 = 0.37

SE of beta1 = 1.700

z = 0.22

Prob > |z| = 0.8263

# Additional File 8. GRADE evidence profile

| Outcome | Comparison | No. of participants  (studies) | SMD with (95% CI) | Risk Ratio (95% CI) | Quality of Evidence | Comments |
| --- | --- | --- | --- | --- | --- | --- |
| Short Term | MT vs Meds | 676 (8 RCTs) | -0.39 (-0.66 to -0.11) | - | Low | Downgraded for high risk of bias and inconsistency |
| Long Term | MT vs Meds | 567 (6 RCTs) | -0.36 (-0.55 to -0.17) | - | Moderate | Downgraded for high risk of bias |
| All-cause Dropouts | MT vs Meds | 745 (8 RCTs) | - | 0.68; 0.52 to 0.90) | Moderate | Downgraded for high risk of bias |
| Adverse Events | MT vs Meds | 426 (5 RCTs) | - | 0.41 (0.12 to 1.42) | Low | Downgraded for high risk of bias and inconsistency |

Guyatt, Oxman, A. D., Akl, E. A., Kunz, R., Vist, G., Brozek, J., Norris, S., Falck-Ytter, Y., Glasziou, P., deBeer, H., Jaeschke, R., Rind, D., Meerpohl, J., Dahm, P., & Schünemann, H. J. (2011). GRADE guidelines: 1. Introduction—GRADE evidence profiles and summary of findings tables. Journal of Clinical Epidemiology, 64(4), 383–394. <https://doi.org/10.1016/j.jclinepi.2010.04.026>

# Additional File 9. Subgroup analysis for short-term pain intensity by treatment component

Forest plot showing the SMD±95% CI for between group comparisons of short-term pain (up to 12 weeks) separate for MT as a standalone treatment and MT plus exercise. The dotted lines represent a between group minimal important difference (MID) threshold of 0.4 SD

# Additional File 10. Subgroup analysis for long-term pain intensity by treatment component

Forest plot showing the SMD±95% CI for between group comparisons of long-term pain (25.8 to 51.6 weeks) separate for MT as a standalone treatment and MT plus exercise. The dotted lines represent a between group minimal important difference (MID) threshold of 0.4 SD

**References**

1. Aboagye E, Lilje S, Bengtsson C, Peterson A, Persson U, Skillgate E. Manual therapy versus advice to stay active for nonspecific back and/or neck pain: a cost-effectiveness analysis. 2022;30(1):27.

2. Afzal R, Ghous M, Shakil Ur Rehman S, Masood T. Comparison between Manual Traction, Manual Opening technique and Combination in Patients with cervical radiculopathy: randomized Control Trial. 2019;69(9):1237‐1241.

3. Alfawaz S, Lohman E, Alameri M, Daher N, Jaber H. Effect of adding stretching to standardized procedures on cervical range of motion, pain, and disability in patients with non-specific mechanical neck pain: a randomized clinical trial. 2020;24(3):50‐58.

4. Arjona Retamal J, Fernández Seijo A, Torres Cintas J, de-la-Llave-Rincón A, Caballero Bragado A. Effects of Instrumental, Manipulative and Soft Tissue Approaches for the Suboccipital Region in Subjects with Chronic Mechanical Neck Pain. A Randomized Controlled Trial. 2021;18(16). Available from: https://www.cochranelibrary.com/central/doi/10.1002/central/CN-02306319/full

5. Ashfaq M, Babur M, Malick W, Hussain M, Awan W. Comparative effectiveness of proprioceptive neuromuscular facilitation and passive vertebral mobilization for neck disability in patients with mechanical neck pain: a randomized controlled trial. 2022;31:16‐21.

6. Ayub A, Osama M, Ahmad S. Effects of active versus passive upper extremity neural mobilization combined with mechanical traction and joint mobilization in females with cervical radiculopathy: a randomized controlled trial. 2019;32(5):725‐730.

7. Barassi G, Supplizi M, Prosperi L, Irace G, Younes A, Della Rovere M, et al. Dual-wavelength high-power laser therapy and neuromuscular manual therapy in chronic neck pain: a randomized clinical trial. 2021;35(2):767‐773.

8. Basson C, Stewart A, Mudzi W. The effect of neural mobilisation on cervico-brachial pain: design of a randomised controlled trial. 2014;15:419.

9. Bautista-Aguirre F, Oliva-Pascual-Vaca Á, Heredia-Rizo A, Boscá-Gandía J, Ricard F, Rodriguez-Blanco C. Effect of cervical vs. thoracic spinal manipulation on peripheral neural features and grip strength in subjects with chronic mechanical neck pain: a randomized controlled trial. 2017;53(3):333‐341.

10. Bevilaqua-Grossi D, Gonçalves M, Carvalho G, Florencio L, Dach F, Speciali J, et al. Additional Effects of a Physical Therapy Protocol on Headache Frequency, Pressure Pain Threshold, and Improvement Perception in Patients With Migraine and Associated Neck Pain: a Randomized Controlled Trial. 2016;97(6):866‐874.

11. Björklund M, Djupsjöbacka M, Svedmark A, Häger C. Effects of tailored neck-shoulder pain treatment based on a decision model guided by clinical assessments and standardized functional tests. A study protocol of a randomized controlled trial. 2012;13:75.

12. Boyles R, Walker M, Young B, Strunce J, Wainner R. The addition of cervical thrust manipulations to a manual physical therapy approach in patients treated for mechanical neck pain: a secondary analysis. 2010;40(3):133‐140.

13. Bronfort G, Evans R, Nelson B, Aker P, Goldsmith C, Vernon H. A randomized clinical trial of exercise and spinal manipulation for patients with chronic neck pain. 2001;26(7):788‐97; discussion 798‐9.

14. Calixtre L, Oliveira A, de Sena Rosa L, Armijo-Olivo S, Visscher C, Alburquerque-Sendín F. Effectiveness of mobilisation of the upper cervical region and craniocervical flexor training on orofacial pain, mandibular function and headache in women with TMD. A randomised, controlled trial. 2019;46(2):109‐119.

15. Casanova-Méndez A, Oliva-Pascual-Vaca A, Rodriguez-Blanco C, Heredia-Rizo A, Gogorza-Arroitaonandia K, Almazán-Campos G. Comparative short-term effects of two thoracic spinal manipulation techniques in subjects with chronic mechanical neck pain: a randomized controlled trial. 2014;19(4):331‐337.

16. Cho J, Lee E, Lee S. Upper cervical and upper thoracic spine mobilization versus deep cervical flexors exercise in individuals with forward head posture: a randomized clinical trial investigating their effectiveness. 2019;32(4):595‐602.

17. Cleland J, Childs J, McRae M, Palmer J, Stowell T. Immediate effects of thoracic manipulation in patients with neck pain: a randomized clinical trial. 2005;10(2):127‐135.

18. Cleland J, Mintken P, Carpenter K, Fritz J, Glynn P, Whitman J, et al. Examination of a clinical prediction rule to identify patients with neck pain likely to benefit from thoracic spine thrust manipulation and a general cervical range of motion exercise: multi-center randomized clinical trial. 2010;90(9):1239‐1250.

19. Corum M, Aydin T, Medin Ceylan C, Kesiktas F. The comparative effects of spinal manipulation, myofascial release and exercise in tension-type headache patients with neck pain: a randomized controlled trial. 2021;43:101319.

20. Cui X, Yao M, Ye X, Wang P, Zhong W, Zhang R, et al. Shi-style cervical manipulations for cervical radiculopathy: a multicenter randomized-controlled clinical trial. 2017;96(31):e7276.

21. de Camargo V, Alburquerque-Sendín F, Bérzin F, Stefanelli V, de Souza D, Fernández-de-las-Peñas C. Immediate effects on electromyographic activity and pressure pain thresholds after a cervical manipulation in mechanical neck pain: a randomized controlled trial. 2011;34(4):211‐220.

22. Desmoulin G, Yasin N, Chen D. Spinal mechanisms of pain control. 2007;23(7):576‐585.

23. Domingues L, Pimentel-Santos F, Cruz E, Sousa A, Santos A, Cordovil A, et al. Is a combined programme of manual therapy and exercise more effective than usual care in patients with non-specific chronic neck pain? A randomized controlled trial. 2019;33(12):1908‐1918.

24. Dunning J, Butts R, Mourad F, Young I, Fernandez-de-Las Peñas C, Hagins M, et al. Upper cervical and upper thoracic manipulation versus mobilization and exercise in patients with cervicogenic headache: a multi-center randomized clinical trial. 2016;17:64.

25. Dziedzic K, Hill J, Lewis M, Sim J, Daniels J, Hay E. Effectiveness of manual therapy or pulsed shortwave diathermy in addition to advice and exercise for neck disorders: a pragmatic randomized controlled trial in physical therapy clinics. 2005;53(2):214‐222.

26. Enthoven P, Skargren E, Oberg B. Clinical course in patients seeking primary care for back or neck pain: a prospective 5-year follow-up of outcome and health care consumption with subgroup analysis. 2004;29(21):2458‐2465.

27. Erdem E, Ünver B, Akbas E, Kinikli G. Immediate effects of thoracic manipulation on cervical joint position sense in individuals with mechanical neck pain: a randomized controlled trial. 2021;34(5):735‐743.

28. Evans R, Bronfort G, Schulz C, Maiers M, Bracha Y, Svendsen K, et al. Supervised exercise with and without spinal manipulation performs similarly and better than home exercise for chronic neck pain: a randomized controlled trial. 2012;37(11):903‐914.

29. Evans R, Bronfort G, Nelson B, Goldsmith C. Two-year follow-up of a randomized clinical trial of spinal manipulation and two types of exercise for patients with chronic neck pain. 2002;27(21):2383‐2389.

30. Evans R, Bronfort G, Bittell S, Anderson A. A pilot study for a randomized clinical trial assessing chiropractic care, medical care, and self-care education for acute and subacute neck pain patients. 2003;26(7):403‐411.

31. Farooq M, Mohseni-Bandpei M, Gilani S, Ashfaq M, Mahmood Q. The effects of neck mobilization in patients with chronic neck pain: a randomized controlled trial. 2018;22(1):24‐31.

32. Galindez-Ibarbengoetxea X, Setuain I, Ramírez-Velez R, Andersen L, González-Izal M, Jauregi A, et al. Short-term effects of manipulative treatment versus a therapeutic home exercise protocol for chronic cervical pain: a randomized clinical trial. 2018;31(1):133‐145.

33. Galindez-Ibarbengoetxea X, Setuain I, Ramírez-Velez R, Andersen L, González-Izal M, Jauregi A, et al. Immediate Effects of Osteopathic Treatment Versus Therapeutic Exercise on Patients With Chronic Cervical Pain. 2018;24(3):24‐32.

34. García-Pérez-Juana D, Fernández-de-Las-Peñas C, Arias-Buría J, Cleland J, Plaza-Manzano G, Ortega-Santiago R. Changes in Cervicocephalic Kinesthetic Sensibility, Widespread Pressure Pain Sensitivity, and Neck Pain After Cervical Thrust Manipulation in Patients With Chronic Mechanical Neck Pain: a Randomized Clinical Trial. 2018;41(7):551‐560.

35. Gattie E, Cleland J, Pandya J, Snodgrass S. Dry Needling Adds No Benefit to the Treatment of Neck Pain: a Sham-Controlled Randomized Clinical Trial With 1-Year Follow-up. 2021;51(1):37‐45.

36. Ghodrati M, Mosallanezhad Z, Shati M, Noroozi M, Moghadam A, Rostami M, et al. Adding Temporomandibular joint treatments to routine physiotherapy for patients with non-specific chronic neck pain: a randomized clinical study. 2020;24(2):202‐212.

37. Gillani S, Ain Q, Rehman S, Masood T. Effects of eccentric muscle energy technique versus static stretching exercises in the management of cervical dysfunction in upper cross syndrome: a randomized control trial. 2020;70(3):394‐398.

38. González Rueda V, López de Celis C, Barra López M, Carrasco Uribarren A, Castillo Tomás S, Hidalgo García C. Effectiveness of a specific manual approach to the suboccipital region in patients with chronic mechanical neck pain and rotation deficit in the upper cervical spine: study protocol for a randomized controlled trial. 2017;18(1):384.

39. González-Iglesias J, Fernández-de-las-Peñas C, Cleland J, Gutiérrez-Vega Mdel R. Thoracic spine manipulation for the management of patients with neck pain: a randomized clinical trial. 2009;39(1):20‐27.

40. González-Rueda V, Hidalgo-García C, Rodríguez-Sanz J, Bueno-Gracia E, Pérez-Bellmunt A, Rodríguez-Rubio P, et al. Does Upper Cervical Manual Therapy Provide Additional Benefit in Disability and Mobility over a Physiotherapy Primary Care Program for Chronic Cervicalgia? A Randomized Controlled Trial. 2020;17(22). Available from: https://www.cochranelibrary.com/central/doi/10.1002/central/CN-02201900/full

41. González-Rueda V, López-de-Celis C, Bueno-Gracia E, Rodríguez-Sanz J, Pérez-Bellmunt A, Barra-López M, et al. Short- and mid-term effects of adding upper cervical manual therapy to a conventional physical therapy program in patients with chronic mechanical neck pain. Randomized controlled clinical trial. 2021;35(3):378‐389.

42. Griswold D, Learman K, Kolber M, O’Halloran B, Cleland J. Pragmatically Applied Cervical and Thoracic Nonthrust Manipulation Versus Thrust Manipulation for Patients With Mechanical Neck Pain: a Multicenter Randomized Clinical Trial. 2018;48(3):137‐145.

43. Groeneweg R, Kropman H, Leopold H, van Assen L, Mulder J, van Tulder M, et al. The effectiveness and cost-evaluation of manual therapy and physical therapy in patients with sub-acute and chronic non specific neck pain. Rationale and design of a Randomized Controlled Trial (RCT). 2010;11:14.

44. Groeneweg R, Haanstra T, Bolman C, Oostendorp R, van Tulder M, Ostelo R. Treatment success in neck pain: the added predictive value of psychosocial variables in addition to clinical variables. 2017;14:44‐52.

45. Groisman S, Malysz T, de Souza da Silva L, Rocha Ribeiro Sanches T, Camargo Bragante K, Locatelli F, et al. Osteopathic manipulative treatment combined with exercise improves pain and disability in individuals with non-specific chronic neck pain: a pragmatic randomized controlled trial. 2020;24(2):189‐195.

46. Gudavalli M, Salsbury S, Vining R, Long C, Corber L, Patwardhan A, et al. Development of an attention-touch control for manual cervical distraction: a pilot randomized clinical trial for patients with neck pain. 2015;16:259.

47. Haas M, Panzer D, Partna L, Lumsden S, Aickin M. Efficacy of cervical endplay assessment as an indicator for spinal manipulation. 2003;28(11):1091‐6; discussion 1096.

48. Haas M, Spegman A, Peterson D, Aickin M, Vavrek D. Dose response and efficacy of spinal manipulation for chronic cervicogenic headache: a pilot randomized controlled trial. 2010;10(2):117‐128.

49. Haas M, Aickin M, Fairweather A, Ganger B, Attwood M, Cummins C, et al. Dose response for chiropractic care of chronic cervicogenic headache and associated neck pain: a randomized pilot study. 2004;27(9):547‐553.

50. Haavik-Taylor H, Murphy B. Cervical spine manipulation alters sensorimotor integration: a somatosensory evoked potential study. 2007;118(2):391‐402.

51. Haleema B, Riaz H. Effects of thoracic spine manipulation on pressure pain sensitivity of rhomboid muscle active trigger points: a randomized controlled trial. 2021;71(7):1720‐1724.

52. Hall T, Chan H, Christensen L, Odenthal B, Wells C, Robinson K. Efficacy of a C1-C2 self-sustained natural apophyseal glide (SNAG) in the management of cervicogenic headache. 2007;37(3):100‐107.

53. Hanney W, Puentedura E, Kolber M, Liu X, Pabian P, Cheatham S. The immediate effects of manual stretching and cervicothoracic junction manipulation on cervical range of motion and upper trapezius pressure pain thresholds. 2017;30(5):1005‐1013.

54. Harrison D, Harrison D, Betz J, Janik T, Holland B, Colloca C, et al. Increasing the cervical lordosis with chiropractic biophysics seated combined extension-compression and transverse load cervical traction with cervical manipulation: nonrandomized clinical control trial. 2003;26(3):139‐151.

55. Harrison D, Cailliet R, Betz J, Haas J, Harrison D, Janik T, et al. Conservative methods for reducing lateral translation postures of the head: a nonrandomized clinical control trial. 2004;41(4):631‐639.

56. Heikkilä H, Johansson M, Wenngren B. Effects of acupuncture, cervical manipulation and NSAID therapy on dizziness and impaired head repositioning of suspected cervical origin: a pilot study. 2000;5(3):151‐157.

57. Holm L, Onell C, Carlseus M, Ekwurtzel R, Holmertz O, Bohman T, et al. Vigorous regular leisure-time physical activity is associated with a clinically important improvement in back pain - a secondary analysis of randomized controlled trials. 2021;22(1):857.

58. Jensen I, Busch H, Bodin L, Hagberg J, Nygren Å, Bergström G. Cost effectiveness of two rehabilitation programmes for neck and back pain patients: a seven year follow-up. 2009;142(3):202‐208.

59. Jordan A, Bendix T, Nielsen H, Hansen F, Høst D, Winkel A. Intensive training, physiotherapy, or manipulation for patients with chronic neck pain. A prospective, single-blinded, randomized clinical trial. 1998;23(3):311‐8; discussion 319.

60. Kanlayanaphotporn R, Chiradejnant A, Vachalathiti R. Immediate effects of the central posteroanterior mobilization technique on pain and range of motion in patients with mechanical neck pain. 2010;32(8):622‐628.

61. Kim S, Kyeong D, Kim M, Kim C, Lee Y, Lee J, et al. The efficacy and safety of non-resistance manual therapy in inpatients with acute neck pain caused by traffic accidents: a randomized controlled trial. 2022;101(22):e29151.

62. Kim D, Chung S, Jung H. The effects of neural mobilization on cervical radiculopathy patients’ pain, disability, ROM, and deep flexor endurance. 2017;30(5):951‐959.

63. Klein R, Bareis A, Schneider A, Linde K. Strain-counterstrain to treat restrictions of the mobility of the cervical spine in patients with neck pain: a sham-controlled randomized trial. 2013;21(1):1‐7.

64. Kongsted A, Qerama E, Kasch H, Bendix T, Bach F, Winther F, et al. Neck collar, “act-as-usual” or active mobilization for whiplash injury? A randomized parallel-group trial. 2007;32(6):618‐626.

65. Korthals-de Bos I, Hoving J, van Tulder M, Rutten-van Mölken M, Adèr H, de Vet H, et al. Cost effectiveness of physiotherapy, manual therapy, and general practitioner care for neck pain: economic evaluation alongside a randomised controlled trial. 2003;326(7395):911.

66. Langenfeld A, Humphreys B, de Bie R, Swanenburg J. Effect of manual versus mechanically assisted manipulations of the thoracic spine in neck pain patients: study protocol of a randomized controlled trial. 2015;16:233.

67. Langevin P, Roy J, Desmeules F. Cervical radiculopathy: study protocol of a randomised clinical trial evaluating the effect of mobilisations and exercises targeting the opening of intervertebral foramen. 2012;13:10.

68. Lascurain-Aguirrebeña I, Newham D, Casado-Zumeta X, Lertxundi A, Critchley D. Immediate effects of cervical mobilisations on neck muscle activity during active neck movements in patients with non-specific neck pain. A double blind placebo controlled trial. 2021;110:42‐53.

69. Leininger B, McDonough C, Evans R, Tosteson T, Tosteson A, Bronfort G. Cost-effectiveness of spinal manipulative therapy, supervised exercise, and home exercise for older adults with chronic neck pain. 2016;16(11):1292‐1304.

70. Leininger B, Evans R, Bronfort G. Exploring patient satisfaction: a secondary analysis of a randomized clinical trial of spinal manipulation, home exercise, and medication for acute and subacute neck pain. 2014;37(8):593‐601.

71. Lewis M, James M, Stokes E, Hill J, Sim J, Hay E, et al. An economic evaluation of three physiotherapy treatments for non-specific neck disorders alongside a randomized trial. 2007;46(11):1701‐1708.

72. Licht P, Christensen H, Højgaard P, Marving J. Vertebral artery flow and spinal manipulation: a randomized, controlled and observer-blinded study. 1998;21(3):141‐144.

73. Lin J, Shen T, Chung R, Chiu T. The effectiveness of Long’s manipulation on patients with chronic mechanical neck pain: a randomized controlled trial. 2013;18(4):308‐315.

74. Lizis P, Kobza W, Manko G, Jaszczur-Nowicki J, Perlinski J, Para B. Cryotherapy With Mobilization Versus Cryotherapy With Mobilization Reinforced With Home Stretching Exercises in Treatment of Chronic Neck Pain: a Randomized Trial. 2020;43(3):197‐205.

75. Lohman E, Pacheco G, Gharibvand L, Daher N, Devore K, Bains G, et al. The immediate effects of cervical spine manipulation on pain and biochemical markers in females with acute non-specific mechanical neck pain: a randomized clinical trial. 2019;27(4):186‐196.

76. López-de-Uralde-Villanueva I, Beltran-Alacreu H, Fernández-Carnero J, La Touche R. Pain management using a multimodal physiotherapy program including a biobehavioral approach for chronic nonspecific neck pain: a randomized controlled trial. 2020;36(1):45‐62.

77. Lopez-Lopez A, Alonso Perez J, González Gutierez J, La Touche R, Lerma Lara S, Izquierdo H, et al. Mobilization versus manipulations versus sustain apophyseal natural glide techniques and interaction with psychological factors for patients with chronic neck pain: randomized controlled trial. 2015;51(2):121‐132.

78. Mahmood T, Afzal W, Ahmad U, Arif M, Ahmad A. Comparative effectiveness of routine physical therapy with and without instrument assisted soft tissue mobilization in patients with neck pain due to upper crossed syndrome. 2021;71(10):2304‐2308.

79. Maiers M, Bronfort G, Evans R, Hartvigsen J, Svendsen K, Bracha Y, et al. Spinal manipulative therapy and exercise for seniors with chronic neck pain. 2014;14(9):1879‐1889.

80. Maiers M, Hartvigsen J, Evans R, Westrom K, Wang Q, Schulz C, et al. Short- or Long-Term Treatment of Spinal Disability in Older Adults With Manipulation and Exercise. 2019;71(11):1516‐1524.

81. Maiers M, Hartvigsen J, Schulz C, Schulz K, Evans R, Bronfort G. Chiropractic and exercise for seniors with low back pain or neck pain: the design of two randomized clinical trials. 2007;8:94.

82. Mansilla-Ferragut P, Fernández-de-Las Peñas C, Alburquerque-Sendín F, Cleland J, Boscá-Gandía J. Immediate effects of atlanto-occipital joint manipulation on active mouth opening and pressure pain sensitivity in women with mechanical neck pain. 2009;32(2):101‐106.

83. Martínez-Segura R, De-La-Llave-Rincón AI, Ortega-Santiago R, Cleland JA, Fernandez-De-Las-Penas C. Immediate changes in widespread pressure pain sensitivity, neck pain, and cervical range of motion after cervical or thoracic thrust manipulation in patients with bilateral chronic mechanical neck pain: a randomized clinical trial. J Orthop Sports Phys Ther. 2012;42(9):806–14.

84. Masaracchio M, Cleland J, Hellman M, Hagins M. Short-term combined effects of thoracic spine thrust manipulation and cervical spine nonthrust manipulation in individuals with mechanical neck pain: a randomized clinical trial. 2013;43(3):118‐127.

85. McDevitt A, Cleland J, Rhon D, Altic R, Courtney D, Glynn P, et al. Thoracic spine thrust manipulation for individuals with cervicogenic headache: a crossover randomized clinical trial. 2022;30(2):78‐95.

86. McReynolds T, Sheridan B. Intramuscular ketorolac versus osteopathic manipulative treatment in the management of acute neck pain in the emergency department: a randomized clinical trial. 2005;105(2):57‐68.

87. Moser N, Mior S, Noseworthy M, Côté P, Wells G, Behr M, et al. Effect of cervical manipulation on vertebral artery and cerebral haemodynamics in patients with chronic neck pain: a crossover randomised controlled trial. 2019;9(5):e025219.

88. Moulson A, Watson T. A preliminary investigation into the relationship between cervical snags and sympathetic nervous system activity in the upper limbs of an asymptomatic population. 2006;11(3):214‐224.

89. Moustafa I, Diab A, Harrison D. The effect of normalizing the sagittal cervical configuration on dizziness, neck pain, and cervicocephalic kinesthetic sensibility: a 1-year randomized controlled study. 2017;53(1):57‐71.

90. Murphy B, Taylor H, Marshall P. The effect of spinal manipulation on the efficacy of a rehabilitation protocol for patients with chronic neck pain: a pilot study. 2010;33(3):168‐177.

91. Nasir M, Jawed R, Baig N, Younus M, Arshad A, Tahir A. A study to observe the effects of physiotherapy with and without manual therapy in the management postural neck pain: a randomized control trial. 2021;71(1(A)):8‐11.

92. NCT00030004. Pilot Study of Spinal Manipulation for Chronic Neck Pain. 2002; Available from: https://www.cochranelibrary.com/central/doi/10.1002/central/CN-01508776/full

93. NCT00269360. Manipulation, Exercise, and Self-Care for Neck Pain. 2005; Available from: https://www.cochranelibrary.com/central/doi/10.1002/central/CN-02013994/full

94. NCT00416117. Manual Physical Therapy and Exercise for Mechanical Neck Disorders. 2006; Available from: https://www.cochranelibrary.com/central/doi/10.1002/central/CN-02013504/full

95. NCT00429624. Randomized Controlled Trial of Chiropractic Manipulation Versus Medical Therapy for Chronic Neck Pain. 2007; Available from: https://www.cochranelibrary.com/central/doi/10.1002/central/CN-01514155/full

96. NCT00713843. Effectiveness of Manual Therapy in Patients With Neck Pain. 2008; Available from: https://www.cochranelibrary.com/central/doi/10.1002/central/CN-02027625/full

97. NCT00978094. Validation of a Novel Sham Cervical Spinal Manipulation Procedure. 2009; Available from: https://www.cochranelibrary.com/central/doi/10.1002/central/CN-01525850/full

98. NCT01161758. Study on the Effect of Cervical Mobilization on Motor Function and Pressure Pain Threshold in Pain Free Individuals. 2010; Available from: https://www.cochranelibrary.com/central/doi/10.1002/central/CN-01530508/full

99. NCT01318720. Short-Term Effects of Combined Manual Therapy to the Cervical and Thoracic Spine. 2011; Available from: https://www.cochranelibrary.com/central/doi/10.1002/central/CN-01532554/full

100. NCT01745705. Cervical Spine Manipulation Affects on Balance and Proprioception. 2012; Available from: https://www.cochranelibrary.com/central/doi/10.1002/central/CN-01539235/full

101. NCT01938209. A Comparison of Seated Thoracic Manipulation and Targeted Supine Thoracic Manipulation on Cervical Flexion Motion and Pain. 2013; Available from: https://www.cochranelibrary.com/central/doi/10.1002/central/CN-01489980/full

102. NCT01962090. Comparison of Two Thoracic Manipulation Techniques to Improve Neck Pain in Dentistry Students. 2013; Available from: https://www.cochranelibrary.com/central/doi/10.1002/central/CN-01536763/full

103. NCT02036905. Cervical and Upper Thoracic Mobilization and Manipulation for Mechanical Neck Pain. 2013; Available from: https://www.cochranelibrary.com/central/doi/10.1002/central/CN-01490605/full

104. NCT02051478. Thoracic Manipulation and Mobilization for Neck Pain. 2014; Available from: https://www.cochranelibrary.com/central/doi/10.1002/central/CN-01543383/full

105. NCT02245425. Comparison of Two Thoracic Manipulation Techniques to Improve Neck Pain. 2014; Available from: https://www.cochranelibrary.com/central/doi/10.1002/central/CN-01548830/full

106. NCT02287337. Validation of CPR for Manipulation for Neck Pain. 2014; Available from: https://www.cochranelibrary.com/central/doi/10.1002/central/CN-01550087/full

107. NCT02301871. The Effect of Soft Tissue Mobilization in Myofascial Neck Pain. 2014; Available from: https://www.cochranelibrary.com/central/doi/10.1002/central/CN-01550523/full

108. NCT02356380. The Effects of Thoracic Spine Mobilizations in Individuals With Neck Pain. 2015; Available from: https://www.cochranelibrary.com/central/doi/10.1002/central/CN-01582739/full

109. NCT02415660. Short-Term Response of Thoracic Spine Manipulation With or Without Trigger Point Dry Needling for Mechanical Neck Pain. 2015; Available from: https://www.cochranelibrary.com/central/doi/10.1002/central/CN-01505702/full

110. NCT02435966. Dry Needling in Patients With Chronic Neck Pain. 2015; Available from: https://www.cochranelibrary.com/central/doi/10.1002/central/CN-01506258/full

111. NCT02552290. Immediate Effects Cervicothoracic Manipulation Versus Passive Upper Trapezius Stretch. 2015; Available from: https://www.cochranelibrary.com/central/doi/10.1002/central/CN-01492312/full

112. NCT02645630. Effects of Pain, Disability and Cervicokinesthesia After Cervical Manipulation. 2015; Available from: https://www.cochranelibrary.com/central/doi/10.1002/central/CN-01554853/full

113. NCT02667821. Vertebral Artery and Cerebral Hemodynamics After Various Head Positions & Manipulation in Patients With Neck Pain. 2016; Available from: https://www.cochranelibrary.com/central/doi/10.1002/central/CN-02037805/full

114. NCT02691143. The Effect of TheraBand® Kinesiology Tape on Post-manipulation Pain and Range of Motion. 2016; Available from: https://www.cochranelibrary.com/central/doi/10.1002/central/CN-01556047/full

115. NCT02731014. Dry Needling for Patients With Neck Pain. 2016; Available from: https://www.cochranelibrary.com/central/doi/10.1002/central/CN-01557148/full

116. NCT02772042. Traction Manipulation of Upper Cervical Spine on Cervicogenic Dizziness. 2016; Available from: https://www.cochranelibrary.com/central/doi/10.1002/central/CN-01558256/full

117. NCT02832232. Specific Manual Approach to the Suboccipital Area on Patients With Chronic Mechanical Neck Pain. 2016; Available from: https://www.cochranelibrary.com/central/doi/10.1002/central/CN-01506796/full

118. NCT02927977. Effectiveness of Dry Needling Adding to Physical Therapy in Patients With Chronic Non-Specific Neck Pain. 2016; Available from: https://www.cochranelibrary.com/central/doi/10.1002/central/CN-01521432/full

119. NCT02972710. A Comparison of Two Thoracic Manipulation Techniques to Improve Neck Pain. 2016; Available from: https://www.cochranelibrary.com/central/doi/10.1002/central/CN-01560188/full

120. NCT02982369. The Effects of Vertebral Manipulation and Pain Education in Chronic Neck Pain. 2016; Available from: https://www.cochranelibrary.com/central/doi/10.1002/central/CN-01593445/full

121. NCT03012503. The Effect of Biofreeze on Post Manipulation Soreness in Patients With Mechanical Neck Pain. 2017; Available from: https://www.cochranelibrary.com/central/doi/10.1002/central/CN-01561079/full

122. NCT03149302. Effects of Local Treatment With and Without Sensorimotor and Balance Exercises in Neck Pain. 2017; Available from: https://www.cochranelibrary.com/central/doi/10.1002/central/CN-02043656/full

123. NCT03157349. Effect of Biofreeze® Versus Placebo on Acute Neck Pain, Disability, and Range of Motion. 2017; Available from: https://www.cochranelibrary.com/central/doi/10.1002/central/CN-01494263/full

124. NCT03176654. The Effect of Manipulation of the Cervical Spine on Pain Biomarkers. 2017; Available from: https://www.cochranelibrary.com/central/doi/10.1002/central/CN-01594712/full

125. NCT03187808. Effects of Single Thoracic Manipulation and Special Massage Technique (RT Technique) on Chronic Mechanical Neck Pain. 2017; Available from: https://www.cochranelibrary.com/central/doi/10.1002/central/CN-01494996/full

126. NCT03190187. Spinal Manipulation Effectiveness in Spinal Disorders. 2017; Available from: https://www.cochranelibrary.com/central/doi/10.1002/central/CN-01588681/full

127. NCT03194490. Benefit of Adding Stretching to Standard Intervention For Patients With Nonspecific Mechanical Neck Pain. 2017; Available from: https://www.cochranelibrary.com/central/doi/10.1002/central/CN-01495135/full

128. NCT03294785. Comparative Effectiveness and Cost-effectiveness of Chuna Manual Therapy for Chronic Neck Pain. 2017; Available from: https://www.cochranelibrary.com/central/doi/10.1002/central/CN-01564419/full

129. NCT03385889. Effects of Cervical Manual Therapy on Cervicogenic Headache. 2017; Available from: https://www.cochranelibrary.com/central/doi/10.1002/central/CN-01567062/full

130. NCT03447977. The Effect of Manual Treatment on Respiratory Parameters, Pain, Posture and Quality of Life in Chronic Neck Pain. 2017; Available from: https://www.cochranelibrary.com/central/doi/10.1002/central/CN-01589539/full

131. NCT03453203. Treatment of Cervical Pain in Chronic Migraine. 2018; Available from: https://www.cochranelibrary.com/central/doi/10.1002/central/CN-01483643/full

132. NCT03507907. The Effects of Mulligan Mobilization Technique in Older Adults With Neck Pain. 2018; Available from: https://www.cochranelibrary.com/central/doi/10.1002/central/CN-02040138/full

133. NCT03509649. Impact of Practitioner and Instructional Set on Subject Perceptions and Expectations of Cervical Spine Manipulation. 2018; Available from: https://www.cochranelibrary.com/central/doi/10.1002/central/CN-01599379/full

134. NCT03560947. Individual Responder Analysis of the Effectiveness of Manual Therapy and Exercise Versus Usual Care in Patients With Chronic Nonspecific Neck Pain. 2018; Available from: https://www.cochranelibrary.com/central/doi/10.1002/central/CN-01609289/full

135. NCT03562338. Predictive Model of Recovery in Patients With Chronic Nonspecific Neck Pain Undergoing Manual Therapy and Exercise. 2018; Available from: https://www.cochranelibrary.com/central/doi/10.1002/central/CN-01660474/full

136. NCT03563079. Effects of Instrument-assisted Soft Tissue Mobilization (IASTM) on Pain and Disability in Individuals With Non-specific Chronic Neck Pain. 2018; Available from: https://www.cochranelibrary.com/central/doi/10.1002/central/CN-01660493/full

137. NCT03903380. Manual Therapy and Exercise With Mixed Reality With Hololens® Exercise Protocol in Chronic Neck Pain Patiens. 2019; Available from: https://www.cochranelibrary.com/central/doi/10.1002/central/CN-01919612/full

138. NCT04054869. Bio-mechanical Reasoning and Lateral Specificity of Upper Cervical Joint Mobilization. 2019; Available from: https://www.cochranelibrary.com/central/doi/10.1002/central/CN-01966404/full

139. NCT04182035. The Effectiveness of Patient-tailored Treatment in Patients With (Sub)Acute Neck Pain. 2019; Available from: https://www.cochranelibrary.com/central/doi/10.1002/central/CN-02010298/full

140. NCT04268667. Comparison of Two Spinal Manipulation Treatments in Patients With Chronic Mechanical Neck Pain. 2020; Available from: https://www.cochranelibrary.com/central/doi/10.1002/central/CN-02072619/full

141. NCT04306640. Impact of Cervical Lordosis Rehabilitation on Autonomic Nervous Function and Cervical Sensorimotor Control. 2020; Available from: https://www.cochranelibrary.com/central/doi/10.1002/central/CN-02089028/full

142. NCT04327739. EFFECTS OF ADDING TWO DIFFERENT TYPES OF MANUAL TECHNIQUES TO A THERAPEUTIC EXERCISE PROGRAM FOR THE MANAGEMENT OF CHRONIC NECK PAIN: A RANDOMIZED CONTROLLED TRIAL OF COMPARATIVE EFFECTIVENESS. 2020; Available from: https://www.cochranelibrary.com/central/doi/10.1002/central/CN-02089487/full

143. NCT04351971. Immediate Effects of the C0-C1 Mobilization Technique in Patients With Chronic Neck Pain. 2020; Available from: https://www.cochranelibrary.com/central/doi/10.1002/central/CN-02180419/full

144. NCT04440293. Effects of Basic Body Awareness Therapy on Patients With Chronic Neck Pain. 2020; Available from: https://www.cochranelibrary.com/central/doi/10.1002/central/CN-02180802/full

145. NCT04455048. The Effectiveness of Manipulation Treatment in Cervical Region. 2020; Available from: https://www.cochranelibrary.com/central/doi/10.1002/central/CN-02134089/full

146. NCT04545996. Sub-Occipital Muscles Inhibition Technique Verses Cranio Cervical Flexion Exercise for Mechanical Neck Pain. 2020; Available from: https://www.cochranelibrary.com/central/doi/10.1002/central/CN-02163588/full

147. NCT04556955. Post Isometric Relaxation Verses Graston Technique in Mechanical Neck Pain. 2020; Available from: https://www.cochranelibrary.com/central/doi/10.1002/central/CN-02181259/full

148. NCT04610255. Dynamic Myofascial Release in Patients With Non-specific Neck Pain. 2020; Available from: https://www.cochranelibrary.com/central/doi/10.1002/central/CN-02197076/full

149. NCT04660292. Clinical Outcomes of Maitland’s Mobilization in Patients With Myofacial Chronic Neck Pain. 2020; Available from: https://www.cochranelibrary.com/central/doi/10.1002/central/CN-02206273/full

150. NCT04702100. Instrument-assisted Soft Tissue Mobilization Versus Integrated on Mechanical Neck Pain. 2021; Available from: https://www.cochranelibrary.com/central/doi/10.1002/central/CN-02233835/full

151. NCT04768790. Effects of Multimodal Exercises Integrated With Cognitive-behavioral Therapy in Subjects With Chronic Neck Pain. 2021; Available from: https://www.cochranelibrary.com/central/doi/10.1002/central/CN-02249257/full

152. NCT04777890. Effects of Instrumental and Manipulative Techniques for the Suboccipital Region in Subjects With Chronic Mechanical Neck Pain. 2021; Available from: https://www.cochranelibrary.com/central/doi/10.1002/central/CN-02249447/full

153. NCT04813315. Effects of Kendall Exercise Versus Gong’s Mobilization in Text Neck Syndrome. A Pilot Study. 2021; Available from: https://www.cochranelibrary.com/central/doi/10.1002/central/CN-02251912/full

154. NCT04856813. Effectiveness of Massage Therapy With Active Component and Therapeutic Exercise in Cervical Pain. 2021; Available from: https://www.cochranelibrary.com/central/doi/10.1002/central/CN-02254846/full

155. NCT04924764. Prediction of Recovery in Patients With Neck Pain. 2021; Available from: https://www.cochranelibrary.com/central/doi/10.1002/central/CN-02278415/full

156. NCT04930575. Muscle Energy Technique Versus Mulligan Technique for Treating Neck Pain in Breast Feeding Women. 2021; Available from: https://www.cochranelibrary.com/central/doi/10.1002/central/CN-02276944/full

157. NCT05004467. Clinical Predictive Effects of Mulligan Treatment in Patients With Chronic Neck Pain. 2021; Available from: https://www.cochranelibrary.com/central/doi/10.1002/central/CN-02297619/full

158. NCT05098860. Efficacy of Exercise, Manual Therapy and Tele-rehabilitation-Assisted Treatment on Degenerative Cervical Diseases. 2021; Available from: https://www.cochranelibrary.com/central/doi/10.1002/central/CN-02341364/full

159. NCT05125250. Effects of Vestibular Exercises and Motor Control in Cervicogenic Dizziness. 2021; Available from: https://www.cochranelibrary.com/central/doi/10.1002/central/CN-02352963/full

160. NCT05186584. Effects of Maitland’s Antero-Posterior Versus Lateral Mobilizations on Cardiovascular Responses. 2022; Available from: https://www.cochranelibrary.com/central/doi/10.1002/central/CN-02366649/full

161. NCT05226559. Effectiveness of Multimodal Physical Therapy in Migraine. 2022; Available from: https://www.cochranelibrary.com/central/doi/10.1002/central/CN-02367451/full

162. NCT05227963. Comparison Between Soft Tissue Mobilization and Strengthening Exercises in Management of Local Neck Syndrome. 2022; Available from: https://www.cochranelibrary.com/central/doi/10.1002/central/CN-02367477/full

163. NCT05257616. Effect of Cervical Mobility on Cardiovascular And Respiratory Outcomes Among Young Adults. 2022; Available from: https://www.cochranelibrary.com/central/doi/10.1002/central/CN-02381962/full

164. NCT05272111. Effects of Fascia Therapy Versus Facial Manipulation on Neck Pain. 2022; Available from: https://www.cochranelibrary.com/central/doi/10.1002/central/CN-02391314/full

165. NCT05308199. Physiotherapy Techniques in Adult Neck Pain. 2022; Available from: https://www.cochranelibrary.com/central/doi/10.1002/central/CN-02385821/full

166. NCT05315076. Comparison of Thoracic Manipulation and Muscle Energy Technique in Non-specific Mechanical Neck Pain. 2022; Available from: https://www.cochranelibrary.com/central/doi/10.1002/central/CN-02392204/full

167. NCT05350254. Implementation of the MAINTAIN Instrument for Patients With Dysfunctional Spinal Pain. 2022; Available from: https://www.cochranelibrary.com/central/doi/10.1002/central/CN-02398565/full

168. NCT05374057. Chiropractic Spinal Manipulative Therapy for Acute Neck Pain. 2022; Available from: https://www.cochranelibrary.com/central/doi/10.1002/central/CN-02395899/full

169. NCT05391997. Effects of Cervical Extension Traction With & Without Modified Cervical and Shoulder Retraction Exercises in Neck Pain. 2022; Available from: https://www.cochranelibrary.com/central/doi/10.1002/central/CN-02405353/full

170. NCT05392465. Effects of Holistic Spinal Fascial Mobilization in Neck Pain. 2022; Available from: https://www.cochranelibrary.com/central/doi/10.1002/central/CN-02405365/full

171. NCT05399953. Investigation of Effects of Physical Therapy Interventions on Mechanical Properties of Muscle After Neck Dissection in Head and Neck Cancer Patients. 2022; Available from: https://www.cochranelibrary.com/central/doi/10.1002/central/CN-02405575/full

172. NCT05404659. Comparative Effects of Oscillatory Mobilizations and Mckenzie Retraction Exercises on Mechanical Neck Pain. 2022; Available from: https://www.cochranelibrary.com/central/doi/10.1002/central/CN-02405689/full

173. NCT05410067. Cervicothoracic Junction Mobilization Versus Eccentric Muscle Energy Technique in Mechanical Cervical Pain. 2022; Available from: https://www.cochranelibrary.com/central/doi/10.1002/central/CN-02402301/full

174. NCT05425706. Sustained Natural Apophysial Glides (SNAGs) Technique in Non-Specific Neck Pain Patients. 2022; Available from: https://www.cochranelibrary.com/central/doi/10.1002/central/CN-02423111/full

175. NCT05474456. Effects of Sustained Natural Apophyseal Glides Versus Cervical Manipulation on Pain and Disability in Wrestle. 2022; Available from: https://www.cochranelibrary.com/central/doi/10.1002/central/CN-02431263/full

176. NCT05496699. The Comparsion of MCkenzie and Mulligan Exercise in Patients With Non-Specific Neck Pain. 2022; Available from: https://www.cochranelibrary.com/central/doi/10.1002/central/CN-02431803/full

177. NCT05502406. Effectiveness of Manual Myofascial Release Versus Instrument Assisted Soft Tissue Mobilization (IASTM) in Patients With Chronic Neck Pain". 2022; Available from: https://www.cochranelibrary.com/central/doi/10.1002/central/CN-02456600/full

178. Nee R, Vicenzino B, Jull G, Cleland J, Coppieters M. Baseline characteristics of patients with nerve-related neck and arm pain predict the likely response to neural tissue management. 2013;43(6):379‐391.

179. Nee R, Vicenzino B, Jull G, Cleland J, Coppieters M. Neural tissue management provides immediate clinically relevant benefits without harmful effects for patients with nerve-related neck and arm pain: a randomised trial. 2012;58(1):23‐31.

180. Ogura T, Tashiro M, Masud M, Watanuki S, Shibuya K, Yamaguchi K, et al. Cerebral metabolic changes in men after chiropractic spinal manipulation for neck pain. 2011;17(6):12‐17.

181. Paanalahti K, Holm L, Nordin M, Asker M, Lyander J, Skillgate E. Adverse events after manual therapy among patients seeking care for neck and/or back pain: a randomized controlled trial. 2014;15:77.

182. Palmgren P, Sandström P, Lundqvist F, Heikkilä H. Improvement after chiropractic care in cervicocephalic kinesthetic sensibility and subjective pain intensity in patients with nontraumatic chronic neck pain. 2006;29(2):100‐106.

183. Park A, Hwang E, Hwang M, Heo I, Park S, Lee J, et al. Cost-Effectiveness of Chuna Manual Therapy and Usual Care, Compared with Usual Care Only for People with Neck Pain following Traffic Accidents: a Multicenter Randomized Controlled Trial. 2021;18(19). Available from: https://www.cochranelibrary.com/central/doi/10.1002/central/CN-02321836/full

184. Peña-Salinas M, Oliva-Pascual-Vaca J, Heredia-Rizo A, Rodriguez-Blanco C, Ricard F, Oliva-Pascual-Vaca Á. No immediate changes on neural and muscular mechanosensitivity after first rib manipulation in subjects with cervical whiplash: a randomized controlled trial. 2017;30(4):921‐928.

185. Pillastrini P, de Lima ESRF, Banchelli F, Burioli A, Di Ciaccio E, Guccione A, et al. Effectiveness of Global Postural Re-education in Patients With Chronic Nonspecific Neck Pain: randomized Controlled Trial. 2016;96(9):1408‐1416.

186. Pillastrini P, Banchelli F, Guccione A, Di Ciaccio E, Violante F, Brugnettini M, et al. Global Postural Reeducation in patients with chronic nonspecific neck pain: cross-over analysis of a randomized controlled trial. 2018;109(1):16‐30.

187. Pires P, Packer A, Dibai-Filho A, Rodrigues-Bigaton D. Immediate and Short-Term Effects of Upper Thoracic Manipulation on Myoelectric Activity of Sternocleidomastoid Muscles in Young Women With Chronic Neck Pain: a Randomized Blind Clinical Trial. 2015;38(8):555‐563.

188. Plaza-Manzano G, Molina-Ortega F, Lomas-Vega R, Martínez-Amat A, Achalandabaso A, Hita-Contreras F. Changes in biochemical markers of pain perception and stress response after spinal manipulation. 2014;44(4):231‐239.

189. Pool J, Ostelo R, Köke A, Bouter L, de Vet H. Comparison of the effectiveness of a behavioural graded activity program and manual therapy in patients with sub-acute neck pain: design of a randomized clinical trial. 2006;11(4):297‐305.

190. Puentedura E, Landers M, Cleland J, Mintken P, Huijbregts P, Fernández-de-Las-Peñas C. Thoracic spine thrust manipulation versus cervical spine thrust manipulation in patients with acute neck pain: a randomized clinical trial. 2011;41(4):208‐220.

191. Puerma-Castillo M, García-Ríos M, Pérez-Gómez M, Aguilar-Ferrándiz M, Peralta-Ramírez M. Effectiveness of kinesio taping in addition to conventional rehabilitation treatment on pain, cervical range of motion and quality of life in patients with neck pain: a randomized controlled trial. 2018;31(3):453‐464.

192. Rampazo É, Telles J, Schiavon M, Liebano R. Hypoalgesic effects of specific vs non-specific cervical manipulation in healthy subjects: a randomized crossover trial. 2021;28:311‐316.

193. Razzaq A, Sajjad A, Yasin S, Tariq R, Ashraf F. Comparison of Cyriax manipulation with traditional physical therapy for the management of cervical discogenic problems. A randomized control trial. 2020;70(8):1329‐1333.

194. Reid S, Rivett D, Katekar M, Callister R. Efficacy of manual therapy treatments for people with cervicogenic dizziness and pain: protocol of a randomised controlled trial. 2012;13:201.

195. Reid S, Rivett D, Katekar M, Callister R. Sustained natural apophyseal glides (SNAGs) are an effective treatment for cervicogenic dizziness. 2008;13(4):357‐366.

196. Rodríguez-Sanz D, Calvo-Lobo C, Unda-Solano F, Sanz-Corbalán I, Romero-Morales C, López-López D. Cervical Lateral Glide Neural Mobilization Is Effective in Treating Cervicobrachial Pain: a Randomized Waiting List Controlled Clinical Trial. 2017;18(12):2492‐2503.

197. Rogers R. The effects of spinal manipulation on cervical kinesthesia in patients with chronic neck pain: a pilot study. 1997;20(2):80‐85.

198. Romero Del Rey R, Saavedra Hernández M, Rodríguez Blanco C, Palomeque Del Cerro L, Alarcón Rodríguez R. Short-term effects of spinal thrust joint manipulation on postural sway in patients with chronic mechanical neck pain: a randomized controlled trial. 2022;44(8):1227‐1233.

199. Rosenfeld M, Seferiadis A, Carlsson J, Gunnarsson R. Active intervention in patients with whiplash-associated disorders improves long-term prognosis: a randomized controlled clinical trial. 2003;28(22):2491‐2498.

200. Ruiz-Sáez M, Fernández-de-las-Peñas C, Blanco C, Martínez-Segura R, García-León R. Changes in pressure pain sensitivity in latent myofascial trigger points in the upper trapezius muscle after a cervical spine manipulation in pain-free subjects. 2007;30(8):578‐583.

201. Saavedra-Hernández M, Castro-Sánchez A, Arroyo-Morales M, Cleland J, Lara-Palomo I, Fernández-de-Las-Peñas C. Short-term effects of kinesio taping versus cervical thrust manipulation in patients with mechanical neck pain: a randomized clinical trial. 2012;42(8):724‐730.

202. Saavedra-Hernández M, Arroyo-Morales M, Cantarero-Villanueva I, Fernández-Lao C, Castro-Sánchez A, Puentedura E, et al. Short-term effects of spinal thrust joint manipulation in patients with chronic neck pain: a randomized clinical trial. 2013;27(6):504‐512.

203. Saayman L, Hay C, Abrahamse H. Chiropractic manipulative therapy and low-level laser therapy in the management of cervical facet dysfunction: a randomized controlled study. 2011;34(3):153‐163.

204. Savva C, Korakakis V, Efstathiou M, Karagiannis C. Cervical traction combined with neural mobilization for patients with cervical radiculopathy: a randomized controlled trial. 2021;26:279‐289.

205. Schwerla F, Bischoff A, Nurnberger A, Genter P, Guillaume J, Resch K. Osteopathic treatment of patients with chronic non-specific neck pain: a randomised controlled trial of efficacy. 2008;15(3):138‐145.

206. Siddiqui M, Akhter S, Baig A. Effects of autogenic and reciprocal inhibition techniques with conventional therapy in mechanical neck pain - a randomized control trial. 2022;23(1):704.

207. Skargren E, Oberg B, Carlsson P, Gade M. Cost and effectiveness analysis of chiropractic and physiotherapy treatment for low back and neck pain. Six-month follow-up. 1997;22(18):2167‐2177.

208. Skillgate E, Vingård E, Alfredsson L. Naprapathic manual therapy or evidence-based care for back and neck pain: a randomized, controlled trial. 2007;23(5):431‐439.

209. Skillgate E, Bohman T, Holm L, Vingård E, Alfredsson L. The long-term effects of naprapathic manual therapy on back and neck pain - results from a pragmatic randomized controlled trial. 2010;11:26.

210. Sparks C, Liu W, Cleland J, Kelly J, Dyer S, Szetela K, et al. Functional Magnetic Resonance Imaging of Cerebral Hemodynamic Responses to Pain Following Thoracic Thrust Manipulation in Individuals With Neck Pain: a Randomized Trial. 2017;40(9):625‐634.

211. Sremakaew M, Jull G, Treleaven J, Barbero M, Falla D, Uthaikhup S. Effects of local treatment with and without sensorimotor and balance exercise in individuals with neck pain: protocol for a randomized controlled trial. 2018;19(1):48.

212. Stieven F, Ferreira G, Wiebusch M, de Araújo F, da Rosa L, Silva M. Dry Needling Combined With Guideline-Based Physical Therapy Provides No Added Benefit in the Management of Chronic Neck Pain: a Randomized Controlled Trial. 2020;50(8):447‐454.

213. van Schalkwyk R, Parkin-Smith G. A clinical trial investigating the possible effect of the supine cervical rotatory manipulation and the supine lateral break manipulation in the treatment of mechanical neck pain: a pilot study. 2000;23(5):324‐331.

214. Vernon H, Triano J, Ross J, Tran S, Soave D, Dinulos M. Validation of a novel sham cervical manipulation procedure. 2012;12(11):1021‐1028.

215. Vernon H, Triano J, Soave D, Dinulos M, Ross K, Tran S. Retention of blinding at follow-up in a randomized clinical study using a sham-control cervical manipulation procedure for neck pain: secondary analyses from a randomized clinical study. 2013;36(8):522‐526.

216. von Piekartz H, Hall T. Orofacial manual therapy improves cervical movement impairment associated with headache and features of temporomandibular dysfunction: a randomized controlled trial. 2013;18(4):345‐350.

217. Walker B, Hebert J, Stomski N, Losco B, French S. Short-term usual chiropractic care for spinal pain: a randomized controlled trial. 2013;38(24):2071‐2078.

218. Williams N, Wilkinson C, Russell I, Edwards R, Hibbs R, Linck P, et al. Randomized osteopathic manipulation study (ROMANS): pragmatic trial for spinal pain in primary care. 2003;20(6):662‐669.

219. Williams N, Edwards R, Linck P, Muntz R, Hibbs R, Wilkinson C, et al. Cost-utility analysis of osteopathy in primary care: results from a pragmatic randomized controlled trial. 2004;21(6):643‐650.

220. Wood T, Colloca C, Matthews R. A pilot randomized clinical trial on the relative effect of instrumental (MFMA) versus manual (HVLA) manipulation in the treatment of cervical spine dysfunction. 2001;24(4):260‐271.

221. Yang L, Lei Z, Jiang J, Zhang L, He C. The therapeutic effect of Neurac training on patients with cervical radiculopathy: a randomized control trial. 2014;45(1):129‐133.

222. Young I, Pozzi F, Dunning J, Linkonis R, Michener L. Immediate and Short-term Effects of Thoracic Spine Manipulation in Patients With Cervical Radiculopathy: a Randomized Controlled Trial. 2019;49(5):299‐309.

223. Yung E, Oh C, Wong M, Grimes J, Barton E, Ali M, et al. Non-thrust cervical manipulations reduce short-term pain and decrease systolic blood pressure during intervention in mechanical neck pain: a randomized clinical trial. 2020;28(2):82‐93.

224. Zaproudina N, Hänninen O, Airaksinen O. Effectiveness of traditional bone setting in chronic neck pain: randomized clinical trial. 2007;30(6):432‐437.
